# IL-10 suppresses T cell expansion while promoting tissue-resident memory cell formation during SARS-CoV-2 infection in rhesus macaques

**DOI:** 10.1371/journal.ppat.1012339

**Published:** 2024-07-01

**Authors:** Christine E. Nelson, Taylor W. Foreman, Eduardo R. Fukutani, Keith D. Kauffman, Shunsuke Sakai, Joel D. Fleegle, Felipe Gomez, NIAID/DIR Tuberculosis Imaging Program, Sydnee T. Gould, Cyril Le Nouën, Xueqiao Liu, Tracey L. Burdette, Nicole L. Garza, Bernard A. P. Lafont, Kelsie Brooks, Cecilia S. Lindestam Arlehamn, Daniela Weiskopf, Alessandro Sette, Heather D. Hickman, Ursula J. Buchholz, Reed F. Johnson, Jason M. Brenchley, James P. Oberman, Artur T. L. Quieroz, Bruno B. Andrade, Laura E. Via, Daniel L. Barber

**Affiliations:** 1 T lymphocyte Biology Section, Laboratory of Parasitic Diseases, National Institute of Allergy and Infectious Disease, National Institutes of Health, Bethesda, Maryland, United States of America; 2 Laboratório de Pesquisa Clínica e Translacional, Instituto Gonçalo Moniz, Fundação Oswaldo Cruz, Salvador, Brazil; 3 Division of Intramural Research, National Institute of Allergy and Infectious Disease, National Institutes of Health, Bethesda, Maryland, United States of America; 4 RNA Viruses Section, Laboratory of Infectious Disease, National Institute of Allergy and Infectious Disease, National Institutes of Health, Bethesda, Maryland, United States of America; 5 SARS-CoV-2 Virology Core, Laboratory of Viral Diseases, National Institute of Allergy and Infectious Diseases, National Institutes of Health, Bethesda, Maryland, United States of America; 6 Barrier Immunity Section, Laboratory of Viral Diseases, National Institute of Allergy and Infectious Disease, National Institutes of Health, Bethesda, Maryland, United States of America; 7 Center for Infectious Disease and Vaccine Research, La Jolla Institute for Immunology, La Jolla, California, United States of America; 8 Department of Medicine, Division of Infectious Diseases and Global Public Health, University of California, San Diego (UCSD), La Jolla, California, United States of America; 9 Viral Immunity and Pathogenesis Unit, Laboratory of Clinical Immunology and Microbiology, National Institute of Allergy and Infectious Disease, National Institutes of Health, Bethesda, Maryland, United States of America; 10 Holy Cross Germantown Hospital, Affiliate of National Breathe Free Sinus and ENT Center, Frederick Breathe Free Sinus and ENT Center, Frederick, Maryland, United States of America; 11 Tuberculosis Research Section, Laboratory of Clinical Infectious Diseases, National Institute of Allergy and Infectious Disease, National Institutes of Health, Bethesda, Maryland, United States of America; 12 Institute of Infectious Disease & Molecular Medicine and Division of Immunology, Department of Pathology, University of Cape Town, Observatory, South Africa; University of Minnesota Twin Cities, UNITED STATES

## Abstract

The regulation of inflammatory responses and pulmonary disease during SARS-CoV-2 infection is incompletely understood. Here we examine the roles of the prototypic pro- and anti-inflammatory cytokines IFNγ and IL-10 using the rhesus macaque model of mild COVID-19. We find that IFNγ drives the development of ^18^fluorodeoxyglucose (FDG)-avid lesions in the lungs as measured by PET/CT imaging but is not required for suppression of viral replication. In contrast, IL-10 limits the duration of acute pulmonary lesions, serum markers of inflammation and the magnitude of virus-specific T cell expansion but does not impair viral clearance. We also show that IL-10 induces the subsequent differentiation of virus-specific effector T cells into CD69^+^CD103^+^ tissue resident memory cells (Trm) in the airways and maintains Trm cells in nasal mucosal surfaces, highlighting an unexpected role for IL-10 in promoting airway memory T cells during SARS-CoV-2 infection of macaques.

## Author summary

Here we examine the roles of the prototypic pro- and anti-inflammatory cytokines IFNγ and IL-10 during SARS-CoV-2 infection of rhesus macaques. Whole body ^18^FDG-PET-CT imaging showed that IFNγ promotes SARS-CoV-2 induced pulmonary disease and IL-10 dampens the size, activity, and duration of lung lesions induced after infection. We also find a major role for IL-10 in the regulation of SARS-CoV-2-specific T cell responses. Our data show that IL-10 limits the magnitude of the effector T cell clonal burst during the acute phase of infection. We also find that following clearance of the virus, IL-10 promotes the differentiation of lung effector T cells into CD69+CD103+ tissue resident memory cells.

## Introduction

SARS-CoV-2 infection has a spectrum of clinical disease outcomes, ranging from asymptomatic to fatal. The severity of COVID-19 is largely determined by the degree of virus-induced damage and immune-mediated pathology [1]. However, the factors that prevent or promote pulmonary inflammation during SARS-CoV-2 infection are not well understood. Rhesus macaques experimentally infected with SARS-CoV-2 develop mild signs of disease and clear most of the virus within a couple weeks [2,3]. Accordingly, this species is a useful model for examining the mechanisms of effective viral control and well-controlled inflammatory response that occurs in most individuals with SARS-CoV-2 infection. Here we use rhesus macaques to examine the roles of prototypic pro- and anti-inflammatory cytokines IFNγ and IL-10, respectively, in host resistance to SARS-CoV-2 infection and the development of COVID-19 disease.

Type I and III interferons have a demonstrated role in control of SARS-CoV-2 infection [4–7]. The contribution of type II IFN, IFNγ, to protection or pathology during COVID-19 is less well understood. IFNγ and molecules induced by IFNγR signaling (e.g., CXCL10) have been associated with severe COVID-19 and the development of acute respiratory distress syndrome [8–18]. Elevated levels of IFNγ also strongly correlate with the development of multi-system inflammatory syndrome in children after SARS-CoV-2 infection [13,19,20]. In ACE2 transgenic mice, neutralizing IFNγ along with TNF reduced mortality of severe SARS-CoV-2 infection [12,21]. However, IFNγ may also contribute to host-protection. IFNγ has been shown to inhibit SARS-CoV-2 replication *in vitro* [22]. Administration of IFNγ to immunocompromised individuals with severe COVID-19 resulted in rapid declines in SARS-CoV-2 viral loads [23]. In the mouse model, IFNγ is required for non-specific protection against SARS-CoV-2 observed after intravenous inoculation with the tuberculosis vaccine Bacillus Calmette–Guérin (BCG) [24,25]. Thus, IFNγ could contribute to protection or lung pathology during SARS-CoV-2 infection depending on the context.

Negative immune regulation also likely also has a key role in determining the outcome of coronavirus infection. In mice, the anti-inflammatory cytokine IL-10 has a protective role in coronavirus induced encephalitis [26–29]. In humans with SARS-CoV-2 infection IL-10 has been associated with severe COVID-19 in multiple studies [10,30,31]. IL-10 is upregulated early in disease progression, and along with IL-6, is a predictive biomarker for poor COVID-19 outcomes [10,30]. However, a study in children reported that higher plasma IL-10 levels were correlated with decreased viral measurements in nasal aspirates [32]. The mechanistic role of IL-10 and IFNγ in the rhesus macaque model of mild SARS-CoV-2 infection has yet to be determined.

In this rhesus macaque model of mild SARS-CoV-2 infection, we find that IL-10 and IFNγ have opposing effects on the development of lung lesions quantified with ^18^FDG-PET/CT imaging without an appreciable effect on SARS-CoV-2 replication. We identify a key role for IL-10 in negatively regulating the clonal expansion of virus-specific CD4 and CD8 T cells. Unexpectedly, we also find that IL-10 drives the differentiation of newly recruited airway effector CD4 and CD8 T cells into tissue resident memory T cells (Trm) after resolution of the infection and has a role in maintaining Trm in the nasal mucosa.

## Results

### ^18^FDG-PET/CT analysis of lung inflammation

**Fig 1:**
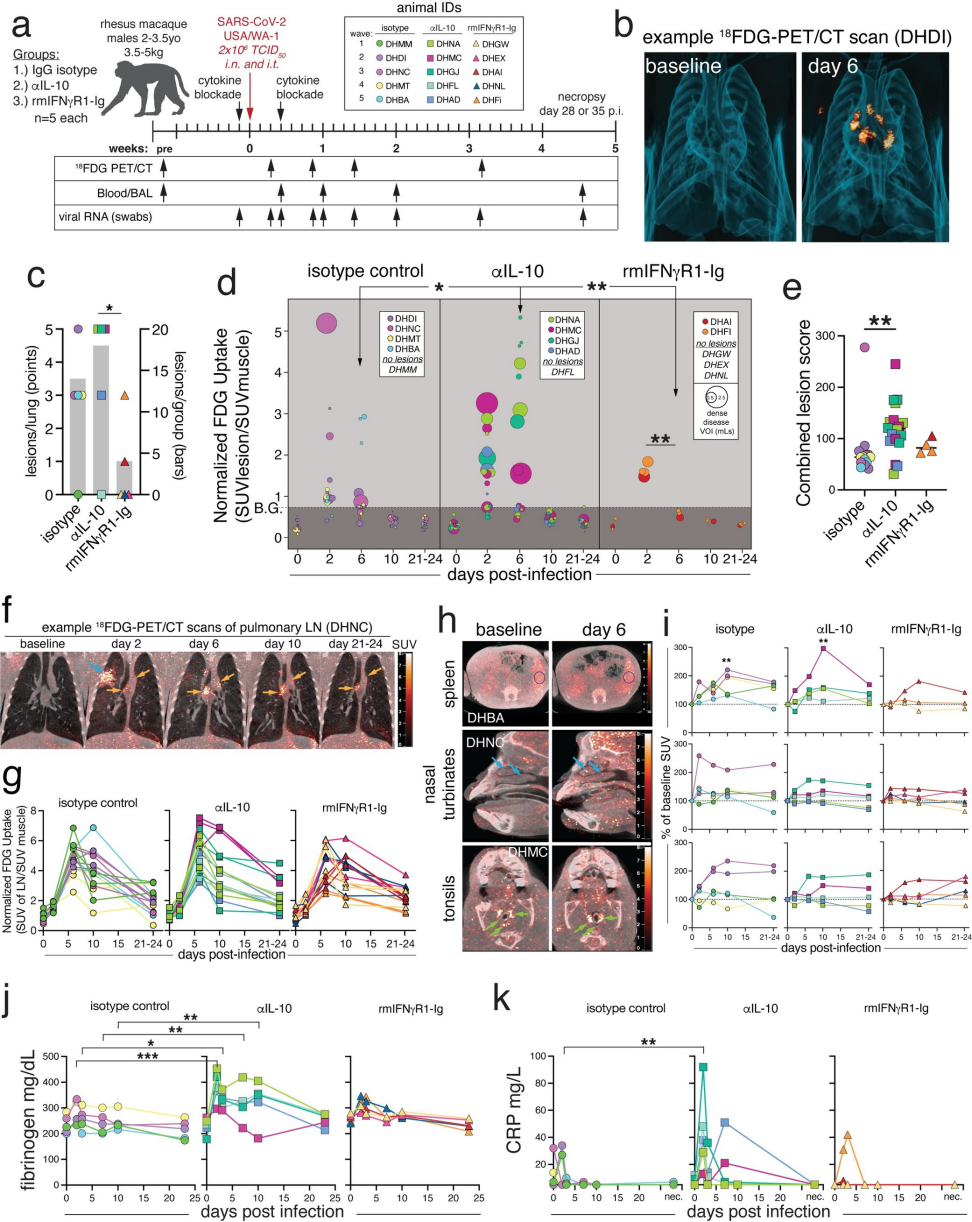
SARS-CoV-2 induced lung inflammation is increased with IL-10 blockade and decreased with IFNγ blockade. (A) Experimental design: Fifteen male rhesus macaques, with n = 5 per group: IgG isotype control, anti-IL-10, or anti-IFNγ (rmIFNγR1-Ig). Animals were treated with 10mg/kg of monoclonal antibody i.v. one day prior to infection and three days after infections with SARS-CoV-2/USA/WA-1 at a dose of 2x10^6^ TCID_50_, administered intranasal (i.n.) and intratracheal (i.t.). Sampling was performed at the indicated timepoints. Individual animal IDs are indicated and used throughout. (B) 3D rendering of representative lung ^18^FDG-PET/CT images from baseline and 6 post infection from isotype control animal (DHDI). (C) Number of lesions per animal (left axis, points) and average number of lesions per group (right axis, grey bars). Significance calculated with individual t-test with Welch’s correction for lesions per animal. (D) Quantification of FDG uptake in standard uptake value (SUV) normalized to muscle, and volume of individual lesions (size of dot), based on volume of interest (VOI) > -550 Hounsfield units (HU) defined at days 2 or 6 post-infection. Significance was calculated with individual t-test with Welch’s correction for FDG uptake at day 6 between groups and Tukey’s multiple comparison test of day 2 vs. day 6 within each group. (E) Lesion score for individual lesions calculated as the sum of normalized max FDG uptake, normalized max Hounsfield’s units, and normalized max volume. Significance calculated with Dunn’s multiple comparison test. (F) Example PET/CT images showing pulmonary lymph node FDG signal from baseline, day 2, 6, 10, and 21–24 post-infection from isotype control animal DHNC. Orange arrows indicate lymph nodes and blue arrow indicates a lung lesion. (G) Quantification of metabolic activity of lymph nodes as measured FDG uptake in SUV, normalized to muscle. All time points post-infection were statistically significant over baseline by 2-way ANOVA and Tukey’s multiple comparison test. (H) Example PET/CT images with evidence of FDG signal from spleen, nasal turbinates, and tonsils from baseline and day 6 post infection. Animal IDs are embedded in image. DHBA and DHNC (isotype). DHMC (anti-IL10). (I) Quantification of change in FDG uptake (SUV) calculated as change from baseline for each animal with detectable signal from spleen, nasal turbinates, and tonsils. Significance calculated by 2-way ANOVA and Tukey’s multiple comparison test. (J) Plasma fibrinogen levels in mg/dL. Significance calculated with 2-way ANOVA and a Dunnett’s multiple comparison test. (K) Plasma C-reactive protein (CRP) in mg/L. Limit of detection >5mg/L. Significance calculated with a 2-way ANOVA and a Dunnett’s multiple comparison test. Panel A generated in part with BioRender.com.

To investigate the role of pro- and anti-inflammatory cytokines in viral replication and pathogenesis during SARS-CoV-2 infection, we treated 15 male rhesus macaques (n = 5/group) with rhesus-modified, effector-silenced (LALA) monoclonal antibody targeting IL-10 (anti-IL-10 IgG1), a rhesus macaque IFNγR1-immunoglobulin fusion protein (rmIFNγR1-Ig), or isotype control (rhesus anti-DSP IgG1) by the intravenous (i.v.) route (Fig 1A). The LALA mutation prevents binding to Fc-gamma receptors and eliminates effector functions against antibody bound targets [33]. One day after treatment, animals were infected with a total of 2x10^6^ TCID_50_ of SARS-CoV-2/USA-WA-1 split between the intranasal (i.n.) and intratracheal (i.t.) routes. An additional dose of blocking reagent was given on day 3 post-infection. Anti-IL-10 and rmIFNγR1-Ig reagents were validated to efficiently block IL-10 and IFNγ signaling *in vitro* using functional cytokine signaling reporter cell lines (S1 Fig). Increased levels of circulating IL-10 after administration of the blocking antibody suggested the formation of antibody/cytokine complexes and persistent drug activity throughout the length of the study. However, increased IFNγ was less apparent in the plasma of animals receiving the IFNγR-Ig fusion reagent (S1 Fig).

We monitored SARS-CoV-2 induced inflammation by ^18^Flurorine deoxyglucose (^18^FDG)-positron emission tomography/computed tomography (PET/CT) imaging of the head, chest, and abdomen. In control animals, we observed evidence of lung inflammation with increased density and ^18^FDG-avidity that peaked at day 2 after SARS-CoV-2 infection and which resolved by days 6–10 post-infection, consistent with previous findings (Figs 1B–1E and S2) [2,34,35]. Lung lesions were primarily peripheral ground glass opacities and consolidations, as well as peri-bronchial consolidations that were ^18^FDG-avid (SUV_mean_ >1.5) [35]. Animals receiving IL-10 blocking antibody had an increased number of lesions that were more metabolically active on day 6 post-infection, as compared to isotype controls or rmIFNγR1-Ig treated animals (Fig 1C and 1D). Each lesion was given an intensity score based on the sum of the normalized maximum values for lesion size, FDG uptake, and density in Hounsfield’s units (HU) (Fig 1E). Lesions from anti-IL-10 treated animals had increased lesion intensity scores compared to controls. At day 6 post-infection when most of the inflammation had resolved in control animals, lesions in the anti-IL-10 treated animals still had significant ^18^FDG uptake and some lesions had increased in intensity from that observed at day 2 post-infection (Fig 1D). Conversely, animals that received rmIFNγR1-Ig tended to have decreased numbers, size, and density of lung lesions as compared to isotype controls or anti-IL-10 treated (Figs 1C–1E and S2). The few lesions that were present in the rmIFNγR1-Ig treated animals were also significantly less dense and metabolically active at day 6 compared to day 2 (Fig 1D). These data suggest that during SARS-CoV-2, IL-10 and IFNγ negatively or positively regulate pulmonary inflammation, respectively.

### ^18^FDG-PET/CT analysis of extra-pulmonary inflammation

We further investigated SARS-CoV-2 induced extra-pulmonary inflammation using ^18^FDG-PET/CT analysis of the pulmonary lymph nodes, spleen, nasal turbinates, and tonsils. ^18^FDG uptake in the pulmonary lymph nodes (pLN) was evident by day 2 post-infection and peaked at day 6–10 post-infection, with some lymph nodes retaining elevated ^18^FDG avidity through days 22–23 post-infection (Figs 1F, 1G and S2). There were no differences between treatment groups in the pLN PET/CT signal after infection (Fig 1G). We also observed increased ^18^FDG uptake in the spleens of isotype control and anti-IL-10 treated animals, that peaked at ~day 10 post-infection and reached statistical significance over baseline (Fig 1H and 1I). However, only 1 of 5 rmIFNγR1-Ig treated animals had splenic ^18^FDG uptake above baseline. The nasal turbinates also had evidence of modest ^18^FDG avidity that peaked at ~day 2–6 post-infection. However, these changes did not reach statistical significance over baseline, and there were no differences between treatment groups. Low levels of ^18^FDG uptake were observed in the tonsils of some animals at ~day 10 post-infection. However, no significant differences were observed between groups.

We also measured circulating soluble markers of inflammation including fibrinogen and C-reactive protein (CRP). Plasma fibrinogen levels were higher in the anti-IL-10 treated animals compared to controls at days 2–10 post-infection, suggestive of increased coagulation (Fig 1J). CRP levels were also elevated in the anti-IL-10 treated animals at day 2 post-infection, as compared to controls (Fig 1K). Collectively, these data suggest that in the rhesus macaque model of mild disease, the lung is the primary site of inflammation following SARS-CoV-2 infection and IL-10 and IFNγ negatively and positively modulated markers of pulmonary inflammation, respectively.

### SARS-CoV-2 replication kinetics and tissue distribution

**Fig 2:**
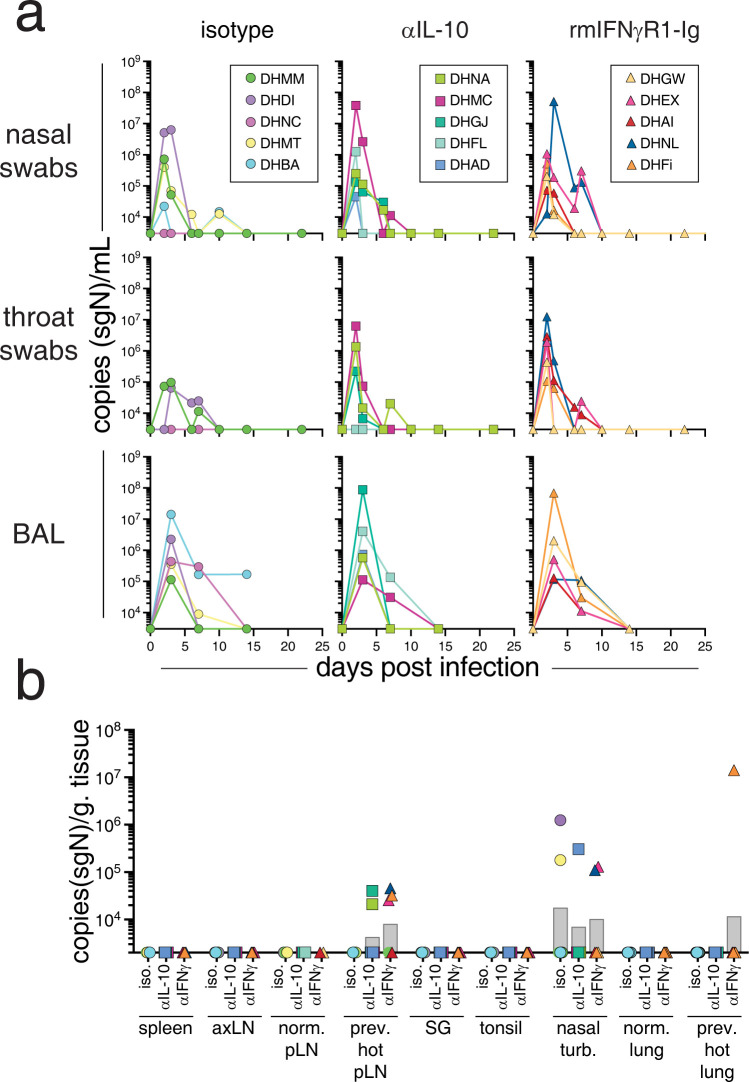
IFNγ and IL-10 are not required to suppress SARS-CoV-2 replication. (A) Subgenomic RNA quantification of the N gene (sgN) of SARS-CoV-2 by RT-qPCR in copies/mL from bronchoalveolar lavage (BAL), nasal swabs, and throat swabs. (B) sgN copies/gram of tissue at necropsy (day 28–35 post-infection) from spleen, axillary lymph node (axLN), non-PET/CT avid pulmonary lymph nodes (norm. pLN), previously PET/CT hot pulmonary lymph nodes (prev. hot pLN), salivary gland (SG), tonsil, nasal turbinates (nasal turb.), normal lung sections (norm. lung), and previously PET/CT hot lung sections. For *A* the cutoff for RNA detection is 3,000 copies/mL. For *B* the cutoff is 2,000 copies/gram of tissue. Graphs show individual animals from samples taken at baseline, days 2, 3, 6, 7, 10, 14, and 22 post-infection, as well as necropsy. Significance calculated with a 2-way ANOVA and a Dunnett’s multiple comparison test.

To determine whether IL-10 or IFNγ blockade influenced SARS-CoV-2 replication, we measured the kinetics of subgenomic RNA of the nucleocapsid gene (sgN) in bronchoalveolar lavage fluid (BAL), nasal swabs, throat swabs, as well as in tissues at necropsy. In the BAL, viral RNA levels were highest at day 3 post-infection and were below the limit of detection (L.O.D.) by days 7–14 (Fig 2A). In nasal and throat swabs, sgN levels peaked at ~2–3 days post-infection and were undetectable in most animals by ~14 days post-infection. We did not observe any statistical difference in viral RNA loads in the BAL or swabs after IL-10 or IFNγ blockade.

At necropsy (day 28 or 35 post-infection), no viral RNA was quantified above the L.O.D. in the spleen, axillary lymph nodes (axLN), pulmonary lymph nodes without previously detected PET/CT signal (norm. pLN), salivary gland (SG), or tonsil (Fig 2B). Viral RNA was detected in previously PET-hot pulmonary lymph nodes (prev. hot pLN) in 2 of 5 anti-IL-10 treated and 3 of 5 rmIFNγR1-Ig, but not in any of the isotype control treated animals; however, the differences between groups did not reach statistical significance. Viral RNA was also detected in the nasal turbinates of a subset of animals, with no differences between groups. Lung sections from previously PET-hot regions were isolated separately from uninvolved lung sections (normal lung). Viral RNA was not detected in any normal lung sections, and in only one of the previously PET-hot lung sections isolated from an rmIFNγR1-Ig treated animal. Altogether, there was little impact of either IL-10 or IFNγ blockade on SARS-CoV-2 peak viral RNA loads or viral RNA clearance in this model, regardless of changes in PET-activity.

### Transcriptional signatures of inflammation in airway immune cells

**Fig 3:**
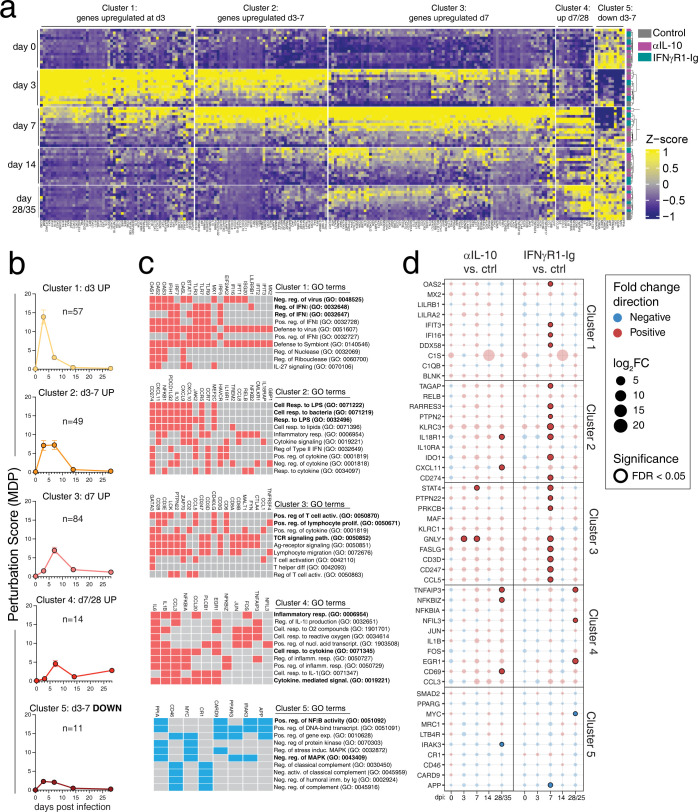
IFNγ blockade prolongs transcriptional signatures of innate and adaptive immune response to SARS-CoV-2 infection. (A) Heatmap of mRNA expression by z-score of BAL immune cells at day 0, 3, 7, 14, and 28–35 after SARS-CoV-2 infection. Genes were pre-filtered on genes that responded to infection, see methods. K-means clustering was used to group genes in to five clusters. (Right) The sample key is based on treatment, grey = control, pink = anti-IL-10, and teal = rmIFNγR1-Ig. (B) Molecular degree of perturbation (MDP) for each gene was calculated based on change from baseline and summarized for each cluster. The number above each graph indicates the number of genes included in each cluster. (C) Gene ontology (GO) classification was performed, and the top significant pathways are shown with the genes included in the pathways highlight in red for genes that are increased over baseline and shown in blue for genes that are downregulated over baseline, identified with EnrichR. Statistical significance reported in S1 Table. (D) The top 10 genes in each cluster were determined and the fold change in each treatment group were compared to controls. The size of the circle represents the fold change, and the color of the circle indicates if the gene is upregulated or downregulated compared to control, at each timepoint. Significant changes are highlighted with bold outline (false discovery rate (FDR) <0.05).

To characterize the impact of IFNγ and IL-10 blockade on immune responses to SARS-CoV-2 infection, we analyzed transcriptional profiles of BAL cells isolated at baseline and days 3, 7, 14 and 28/35 after infection using Nanostring transcriptomics (n = 752 immune response genes). To isolate genes that specifically responded to infection, we calculated the molecular degree of perturbation (MDP) for each gene with day 0 as a reference, and genes with an MDP score <2 were considered low variability and >2 considered moderately or highly variable [36]. Each gene was then given a variability score based on the *three period’s classification mean* across all timepoints, and only genes with a score of ≥1.2 were classified as variable from baseline in response to SARS-CoV-2 infection and included in downstream analysis. Groups of SARS-CoV-2 responsive genes were defined by k-means clustering of mRNA expression data (Fig 3A). This clustering revealed 5 distinct groups of kinetically co-regulated genes (Fig 3A and 3B). Cluster 1 was comprised of genes that were highly upregulated at day 3 following infection and rapidly declined thereafter. Cluster 2 contained genes that were upregulated through days 3–7 post-infection. Cluster 3 were genes that clearly peaked at day 7 and declined rapidly after. Cluster 4 genes were elevated from day 7 onward. Lastly, cluster 5 contained genes that were downregulated after infection (Fig 3A and 3B).

Gene ontology (GO) enrichment analysis classified the gene clusters based on discrete immunological pathways [37]. Cluster 1 genes, which were upregulated at day 3 post-infection, were highly enriched for genes that respond to viral infection and are involved in the regulation of type I IFN responses (Fig 3C, S1 Table). Genes involved in viral RNA sensing, like TLRs 3/7/9 and IRF5 were upregulated in cluster 1, as were interferon stimulated genes, like OAS1/2/3, IFIT1/2/3, IFI16, and MX1/2 (Fig 3C). Cluster 2, which represented genes up at days 3–7 post-infection, was defined by broadly inflammatory genes that are upregulated in respond to infection, LPS, and bacteria (Fig 3C). CXCL9/10/11, NFKB1, CD274 (PD-L1), IDO1, and IL10 were all elevated at days 3–7 post-infection in cluster 2 (Fig 3A and 3C). Cluster 3, which identified genes upregulated at day 7 post-infection, was highly enriched for T cell responsive genes, including those involved in T cell activation, lymphocyte proliferation, and TCR signaling, e.g. CD3D/E/G, CD8A/B, CD28, CD40LG, and GATA3 (Fig 3C). Genes involved in T/NK cell cytotoxicity, (e.g. GZMB, GZMK, PRF1, FASLG, KLRD1, and KLRC1) were also highly upregulated at day 7 in cluster 3 (Fig 3A). Cluster 4 had a limited set of genes, n = 14, that was upregulated at from day 7 onwards and was enriched for cellular responses to cytokines and included IL1B, IL6, IL8, FOS, and JUN (Fig 3A and 3C). Cluster 5, which represented a small number of genes downregulated from baseline after infection (e.g. IRAK3, MRC1, MYC, APP, and PPARG) and may indicate a loss of alveolar macrophages, which has been previously described for SARS-CoV-2 infection (Figs 3A and S3) [2].

To identify differences between treatment groups within each gene cluster, the top 10 genes defining each gene cluster were identified and differential expression was determined based on fold change over time for each treatment and then compared to control. Several genes across clusters 1, 2, and 3 were upregulated with IFNγ blockade compared to isotype controls at day 7 (Fig 3D). In cluster 1, OAS2, IFIT3, IFI16, and DDX58 were higher in the rmIFNγR1-Ig treated animals at day 7, despite this cluster of genes being defined as genes that are highly induced at day 3 post-infection (Figs 3D and S3). This may suggest a prolonged type I IFN signature with IFNγ blockade. Cluster 2 genes also had a differential pattern of expression with IFNγ blockade. For example, cluster 2 genes were upregulated at day 3 post-infection and began to decline by day 7 in control and anti-IL-10 treated animals. With IFNγ blockade, genes in cluster 2, including IL18R1, IDO1, CXCL11, CD274 (PD-L1), and PTPN2, were similarly expressed at day 3 post-infection but were more highly expressed at day 7 compared to controls and had increased expression over the day 3 timepoint (S3 Fig). Together, these data suggest a prolonged innate inflammatory response to SARS-CoV-2 with IFNγ blockade. Genes in cluster 3 were also upregulated in the IFNγ blockade group at day 7 and included genes suggestive of T cell activation, e.g. CD3D, CD247, STAT4, PTPN22, and GRLY. A small number of genes in cluster 3 were upregulated in anti-IL-10 treated animals compared to controls, including STAT4 and GNLY. Overall, most changes in gene expression were observed after IFNγ blockade on day 7 and suggested prolonged innate inflammation and increased adaptive immune responses.

### Innate lymphocyte responses

NK cells are important in early control of viral infections and have been shown to produce IFNγ in response to SARS-CoV-2 [38]. We investigated NK cells response in the peripheral blood mononuclear cell (PBMC) compartment and BAL. In NHP, NK cells can be defined as CD3^-^/CD8α^+^/CD8β^-^/NKG2A^+^ and divided into 4 distinct subpopulations based on the expression of CD16 and CD56 [39–42]. The population of CD16^+^/CD56^-^ NK cells have been shown to be cytotoxic, with the expression of perforin and granzyme B [39]. CD16^-^/CD56^+^ NK cells are thought to be less cytotoxic and more likely to produce IFNγ [41]. In PBMCs, we observed a skewing of the NK population to a predominately CD16^+^/CD56^-^ cytotoxic phenotype, which is consistent with previous reports (S4A and S4B Fig) [40]. In the BAL, CD16^-^/CD56^+^ cytokine producing NK cells were the most abundant. After SARS-CoV-2 infection, there was an increase in total NK cells that peaked at day 7 in isotype control samples in both the PBMCs and BAL (S4B Fig). All NK cell subsets in PMBCs had upregulated Ki67, a marker of cell-cycle/proliferation, by day 7 post-infection (S4C and S4D Fig). Ki67 upregulation was less robust in the BAL than in PBMCs. Granzyme B was differentially expressed by NK cell subsets, with CD16^+^/CD56^-^ in both the PBMC and BAL having the highest expression of granzyme B (S4E and S4F Fig). All NK subsets in both PBMC and BAL upregulated granzyme B at day 3 in response to SARS-CoV-2 infection and had mostly returned to baseline levels by 4–5 weeks post-infection. Neither IFNγ nor IL-10 blockade had a substantial impact on the expansion of NK cells or their function after infection. These data suggest that NK cells in the blood and lungs respond to SARS-CoV-2 infection but are not highly dependent on IL-10 or IFNγ.

Mucosal Associated Invariant T cells (MAIT cells) are innate-like lymphocytes that recognize 5-OP-RU, a small molecule produced during microbial riboflavin biosynthesis, presented by the MHC-I-like molecule MR1 [43]. In the context of viral infections, MAIT cells have been shown to be host protective via cytokine-driven, MR1-independent mechanisms [44–47]. MAIT cells have also been suggested to play a role in SARS-CoV-2 infection [48,49], so we next investigated the MAIT cell responses to SARS-CoV-2 in PBMCs and BAL. We observed an increase in Ki67 expression by MAITs in PBMCs at day 7 post-infection (S5A and S5B Fig). However, MAIT cell frequencies in PBMCs and BAL were relatively stable after SARS-CoV-2 infection (S5C Fig). At necropsy, we assessed MAIT cells in the spleen, lymph nodes, tonsil, and lung. We did not observe any changes in MAIT cell frequency in any compartment with either IL-10 or IFNγ blockade. However, there was an increase in Ki67^+^ MAIT cells in the PBMC and BAL on day 7 in the anti-IL-10 treated animals relative to controls (S5C Fig). These data suggest that, in rhesus macaques, MAIT cells become activated in response to SARS-CoV-2 but do not significantly expand in frequency. Moreover, IL-10 has a minor role in inhibiting MAIT cell proliferation during SARS-CoV-2 infection.

### B cells and antibody responses

Circulating anti-spike antibodies are correlated with protection against symptomatic SARS-CoV-2 infection provided by vaccines [50–53]. We investigated whether IL-10 or IFNγ blockade resulted in changes to B cells and SARS-CoV-2 specific antibody responses. The frequency of total B cells in PBMCs remained unchanged after SARS-CoV-2 infection in all treatment groups (S6 Fig). In the BAL, there was a statistically significant, albeit small, increase in total B cells at day 3 in the rmIFNγR1-Ig treatment group compared to isotype controls. At necropsy, there were no differences in the frequency of total B cells in tissue between treatment groups (S6 Fig). However, germinal center B cells (CD20^+^/BCL6^+^/Ki67^+^) were decreased in the previously PET-hot pLNs with IFNγ blockade (S6D Fig).

Anti-spike and anti-RBD antibodies were detected in the plasma of all animals beginning as early as day 7 post-infection (S6E Fig). Virus-specific IgM responses peaked on ~day 14 post-infection and began to plateau or decline by day 28–35. Virus-specific IgG and IgA levels continued to increase throughout the length of the study and had not yet reached a plateau at the day 28–35 endpoint. Anti-IL-10 treated animals had higher levels of S-specific IgG and IgA on day 7 relative to the controls. However, by day 14 this difference was no longer significant, and overall, there were no substantial changes to the antibody responses with either IL-10 or IFNγ blockade. SARS-CoV-2 neutralizing antibodies were detected in 14 of 15 animals, apart from control animal DHMT, which also had the lowest anti-spike and RBD antibody titers at necropsy for all isotypes (S6F Fig). Plasma anti-RBD IgG and IgA levels correlated with live-virus neutralization titers at necropsy (S6G Fig). There were no statistically significant differences in neutralization titers between groups. Altogether, these data show that SARS-CoV-2-specific antibody responses are first detectable around day 7 and continue to increase over the first month of infection. While IFNγ may play a role in promoting GC responses in reactive pLNs, it has no major impact on the final development of serum antibody responses to SARS-CoV-2 infection in the observed timeframe. Additionally, while IL-10 blockade may accelerate the induction of serum antibody responses, it did not lead to sustained increases in SARS-CoV-2 specific antibody responses or increases in neutralizing antibody titers.

### Kinetics of SARS-CoV-2 specific T cell responses in the BAL and blood

**Fig 4:**
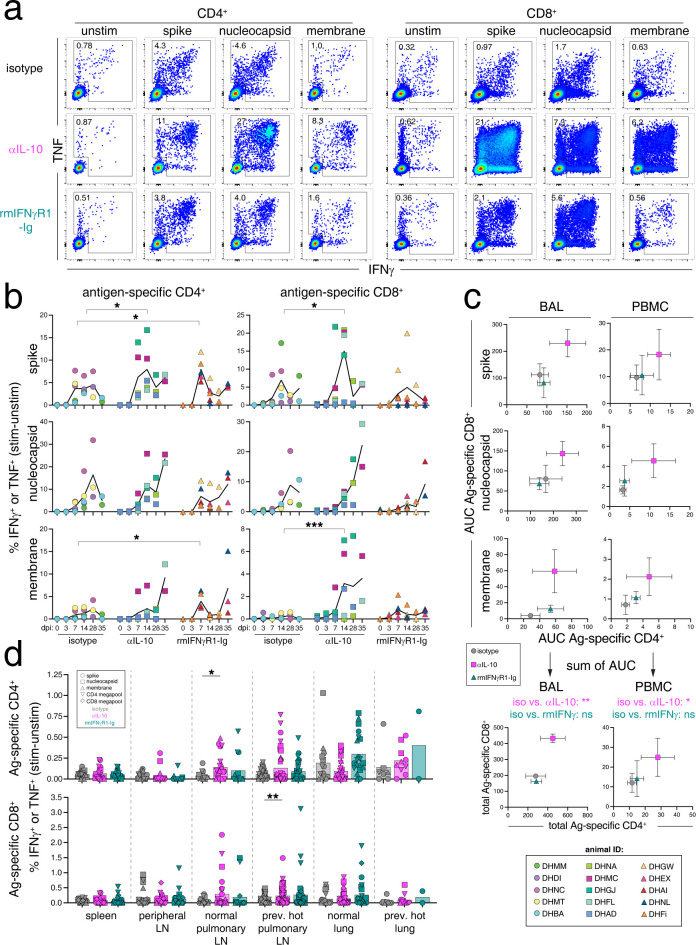
IL-10 blockade increases SARS-CoV-2-specific T cell responses in the blood and BAL fluid. (A) Representative flow cytometry plots of CD4^+^95^+^ and CD8^+^95^+^ T cells from the bronchoalveolar lavage (BAL) at day 14 post-infection responding to *ex vivo* peptide stimulation assay with SARS-CoV-2 15-mer peptide pools for spike (S), nucleocapsid (N), and membrane (M) proteins by production of IFNγ and TNF production. Numbers in plots are the frequency of the gated cytokine+ population. (B) Quantification of frequency of antigen specific CD4^+^95^+^ and CD8^+^95^+^ responses in BAL at baseline (dpi 0), days 3, 7, 14, and necropsy (dpi 28 or 35), calculated by taking the frequency of IFNγ^+^ or TNF^+^ in the stimulated samples and subtracting the frequency in the matched unstimulated samples. Each animal is represented as a point and the mean as a line for each treatment group. Legend is in bottom right corner. Significance calculated by 2-way ANOVA with Dunnett’s multiple comparison test. (C) The mean and SEM of the area under the curve (AUC) for antigen-specific CD4 T cell responses (x-axis) and antigen-specific CD8 T cell responses (y-axis) responses in BAL and PBMC samples calculated from *ex vivo* peptide stimulation with spike (S), nucleocapsid (N), and membrane (M), as represented in *B*. The AUC was determined for dpi 0–28 and interpolated by linear regression for animals necropsied at day 35. The bottom graphs represent the sum of the AUC for the S-, N-, and M-specific CD4 and CD8 T cell responses, and statistics represent a Dunnett’s multiple comparison test for total AUC responses from treatment groups compared to isotype control. (D) Quantification of frequency of antigen specific CD4^+^95^+^ and CD8^+^95^+^ responses in spleen, peripheral lymph nodes (axillary, cervical, and/or inguinal lymph nodes), normal pulmonary lymph nodes (norm. pulm. LN), previously PET/CT hot pulmonary lymph nodes (prev. hot pulm. LN), normal lung sections (norm. lung), and previously PET/CT hot lung sections (prev. hot lung) at necropsy (dpi 28 or 35), calculated as in *B*. Each animal is represented as a point and the antigen as a shape. Significance calculated by 2-way ANOVA with Dunnett’s multiple comparison test.

T cell responses likely have an important role in protection against SARS-CoV-2 infection in humans [54]. The kinetics of CD4 and CD8 T cells that recognize the major SARS-CoV-2 structural protein antigens of spike (S), nucleocapsid (N), and membrane (M) were measured by *ex vivo* peptide re-stimulation of the PBMCs and BAL cells. Consistent with our previous findings, SARS-CoV-2-specific T cell responses were first detected at day 7 post-infection in both the PBMCs and BAL. The S and N epitopes were immunodominant compared to M, and the frequency of virus-specific T cells in the BAL was ~5–50 fold higher as compared to the blood (Fig 4A–4C) [2]. SARS-CoV-2-specific CD4 and CD8 T cell responses were increased with IL-10 blockade, as compared to controls (Fig 4B). The area under the curve for the S-, N- and M-specific CD4 and CD8 T cell responses from each animal in PBMCs and BAL was calculated. The combined S-, N- and M-specific responses were summed, and the cumulative SARS-CoV-2-specific CD4 and CD8 T cell responses in BAL and PBMCs were significantly elevated with IL-10 blockade but were unchanged with IFNγ blockade, as compared to controls (Fig 4C). These data suggest that IL-10 limits early Ag-specific CD4 and CD8 T cell responses to SARS-CoV-2. These data highlight the need to quantify Ag-specific T cell response by flow cytometry, rather than relying solely on bulk transcriptomics.

At necropsy, T cells responding to S, N, M and SARS-CoV-2 peptide megapools [55] were assessed in tissues. Low frequencies of Ag-specific CD4 and CD8 T cells were detected in the spleen and lymph nodes, with previously hot pLNs having the largest relative responses among the secondary lymphoid organs examined (Fig 4D). Normal and previously PET-hot lung specimens also had detectable populations of Ag-specific CD4 and CD8 T cells. We observed a small but statistically significant increase in the frequency of Ag-specific T cells in pLNs after anti-IL-10 treatment, with Ag-specific CD4 T cells in normal pLNs and Ag-specific CD8 T cells in previously hot pLNs both being increased compared to isotype control. These data are consistent with the elevated virus-specific CD4 and CD8 T cell responses observed in the BAL after IL-10 blockade. Furthermore, these data suggest that IFNγ has little role in regulating the expansion of SARS-CoV-2-specific T cells or their migration into the lungs and lower airways despite evidence of increases in T cell responses by transcriptomic analysis.

### T cell responses in the nasal mucosa

**Fig 5:**
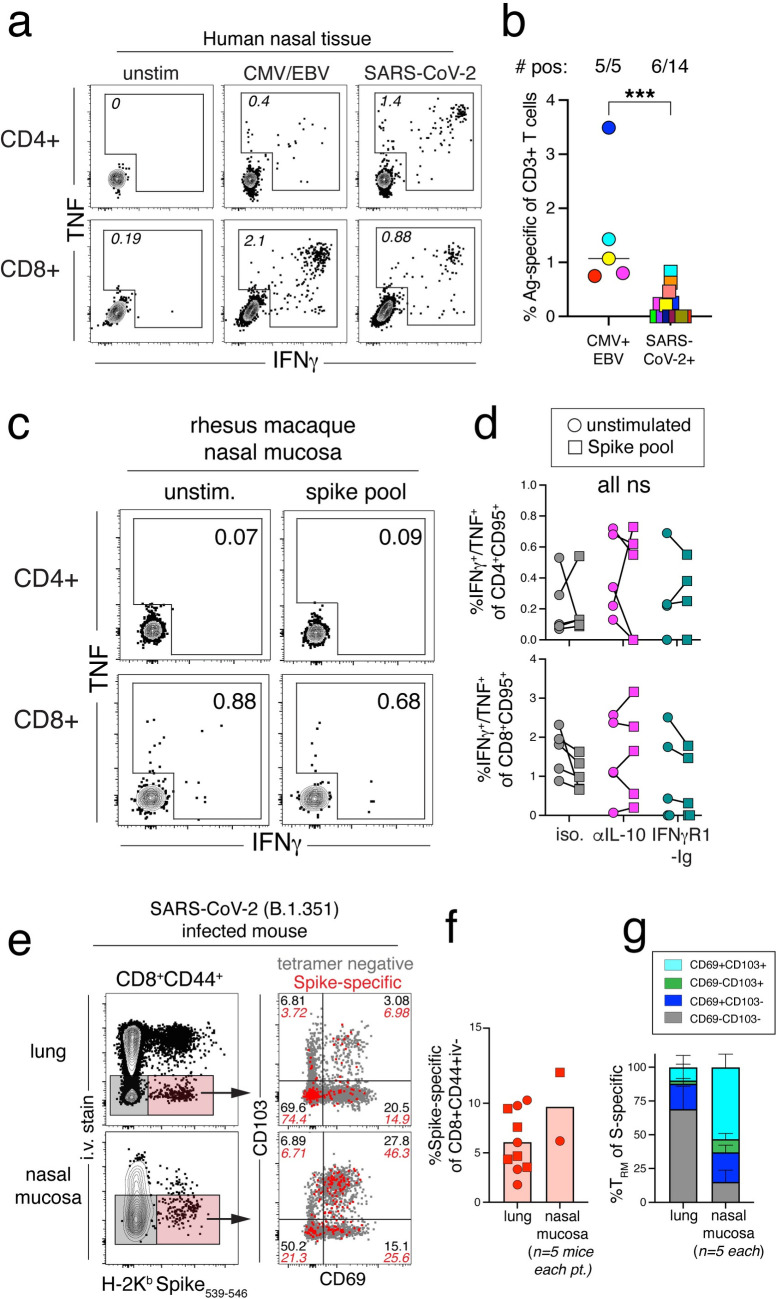
IL-10 blockade does not rescue the lack of SARS-CoV-2-specific T cell responses in the nasal mucosa of rhesus macaques. (A) Representative flow cytometry plots of cytokine producing CD4^+^95^+^ or CD8^+^95^+^ T cells after ex vivo peptide stimulation with CMV and EBV peptide pools, SARS-CoV-2 peptide pools, or unstimulated samples from human nasal mucosa. Numbers in plots are the frequency of gated cytokine+ of activated CD4 or CD8 T cells. (B) Quantification of total antigen-specific T cells by cytokine+ (IFNγ+/TNF+) of total CD3+ T cells after stimulation with the indicated peptide pools. Number of samples with positive signal above background were calculated by subtracting the total cytokine+ T cells response in the unstimulated samples from the total cytokine+ response in the stimulated samples. Significance calculated with unpaired t-test. (C) Representative flow cytometry plots of CD4^+^95^+^ or CD8^+^95^+^ T cells responding to spike peptide pool from the nasal mucosa of isotype control rhesus macaque, DHDI, at necropsy (dpi 28). Numbers in plots are the frequency of the gated cytokine+ in stimulated or unstimulated samples. (D) Quantification of frequency of cytokine+ (IFNγ+ and/or TNF+) CD4^+^95^+^ or CD8^+^95^+^ T cells responding to spike peptide stimulation at necropsy (dpi 28 or 35) from stimulated and unstimulated samples from the nasal mucosa. Significance calculated with a 2-way ANOVA and Sidak’s multiple comparison test between stimulated and unstimulated samples. (E) (*Left*) Representative flow cytometry plots of i.v. stain and Spike tetramer (H-2K^b^ Spike_539-546_) CD8^+^CD44^+^ T cells from the lung and nasal mucosa of mice infected with SARS-CoV-2 (B.1.351) at necropsy (dpi 30). Red shaded gate shows parenchymal (i.v. negative) Spike-specific CD8 T cells and grey shaded gate represents parenchymal (i.v. negative) non-antigen-specific CD8 T cells. (*Right*) Representative flow cytometry plots of CD69 and CD103 expression by parenchymal (i.v. negative) spike-specific (red dots) and non-specific (grey dots) CD8 T cells from the lung and nasal mucosa. Numbers in plots indicate the frequency within the quadrants. (F) Quantification of the frequency of parenchymal (i.v. negative) spike-specific CD8 T cells from the lung and nasal mucosa from mice infected with SARS-CoV-2 (B.1.351) at necropsy (dpi 30). Data shown from two separate experiments, squares represent one experiment and circles represent a second experiment. Nasal mucosa samples were pooled (n = 5) prior to staining and each experiment represented as one data point. (G) Quantification of the frequency of CD69^-^CD103^-^ (grey bars), CD69^+^CD103^-^ (dark blue bars), CD69^-^CD103^+^ (green bars), or CD69^+^CD103^+^ (turquoise bars), of parenchymal spike-specific CD8 T cells from the lungs and nasal mucosa of mice infected with SARS-CoV-2 (B.1.351) at necropsy (dpi 30).

Immunity in the nasal mucosa is likely critical in protection from SARS-CoV-2 (re)infection in humans [56]. Previous work from Lim et al. showed that SARS-CoV-2 specific T cells can be detected in the nasal mucosa after breakthrough infection in previously vaccinated individuals [57]. However, in our previous work we were unable to detect SARS-CoV-2 specific T cells in the nasal mucosa of intranasally infected macaques [2]. To evaluate SARS-CoV-2-specific T cells in the human nasal mucosa, we obtained freshly resected samples of frontal, ethmoid, or maxillary sinuses from individuals undergoing surgical resection for inflammatory conditions unrelated to SARS-CoV-2 infection. Lymphocytes extracted from the nasal mucosa were restimulated with overlapping peptide pools derived from SARS-CoV-2 nucleocapsid and spike proteins or from CMV and EBV, as a positive control. SARS-CoV-2 specific CD4 or CD8 T cell responses were detected in 43% (6/14) of samples (Fig 5A). In comparison, we detected CMV/EBV-specific T cells in 100% (5/5) of samples, and frequencies tended to be higher than those observed for SARS-CoV-2 specific T cells. While the SARS-CoV-2 vaccination status and infectious history of the de-identified individuals is unknown in this study, it confirms that SARS-CoV-2 specific T cells in humans can reach the nasal mucosa.

Given the discrepancy in the detection of SARS-CoV-2 specific T cells in humans versus macaques, we next asked if our IL-10 blockade regimen increased T cell responses in the nasal mucosa of infected macaques to detectable levels. We isolated the epithelial lining of the nasal passage, including the nasal turbinates, at 4–5 weeks post-infection and restimulated the extracted lymphocytes with the spike (S) peptide pool. S-specific T cells were not detected in the nasal mucosa of control animals, consistent with previous findings in this model (Fig 5C and 5D) [2]. Moreover, none of the animals receiving IL-10 or IFNγ blockade animals had detectable virus-specific T cells above baseline in the nasal mucosa. These data indicate that the lack of nasal SARS-CoV-2 specific T cells in macaques is not due to IL-10 mediated suppression.

We next asked if the absence of SARS-CoV-2-specific T cell responses in the nasal mucosa is unique to rhesus macaques by examining responses in the nasal mucosa of SARS-CoV-2 infected mice. C57BL/6 mice were intranasally infected with SARS-CoV-2 beta strain (B.1.351). At necropsy, intravenous antibody labelling prior to necropsy was used to identify cells in the tissue vasculature. Virus-specific CD8 T cell responses in the nasal mucosa and lungs were quantified with K^b^/Spike_539-546_ tetramer. At day 30 post-infection, parenchymal spike-specific CD8 T cells were detected in both the lung and nasal mucosa (Fig 5E and 5F). SARS-CoV-2-specific T cells in the nasal mucosa had a highly tissue resident memory (Trm) phenotype, with ~50% of spike-specific T cell CD69^+^CD103^+^, compared to ~10% in the lung (Fig 5G). Therefore, SARS-CoV-2 infection generates spike-specific Trm in the nasal mucosa of mice and a subset of patients, but virus-specific responses are not detectable in the rhesus macaque model.

To determine if rhesus macaques could generate Ag-specific T cells in the nasal mucosa in response to another pulmonary infection, we assessed T cells from the nasal mucosa of *Mycobacterium tuberculosis* (Mtb) infected animals. Three male rhesus macaques were infected with ~50 CFU of Mtb-H37Rv via endobronchial instillation, and Mtb-specific T cells from the nasal mucosa were quantified by intracellular cytokine staining after restimulation with Mtb peptide pools [58,59]. Mtb-specific CD4 and CD8 T cells were readily detected in the nasal mucosa of all infected rhesus macaques and ~50–65% of the IFNγ^+^TNF^+^ CD4 and CD8 T cells expressed CD69 (S7 Fig).

Thus, SARS-CoV-2 specific nasal mucosa T cells are detected in humans and mice but not in macaques. This difference cannot be attributed to technical limitations of restimulation of nasal lymphocytes in macaques, as Mtb-specific T cells are readily detected in the nasal mucosa after infection. Moreover, IL-10 blockade boosted the clonal burst of virus-specific CD4 and CD8 T cells in the airways yet did not expand CD4 and CD8 T cells in the nasal mucosa to detectable levels.

### Development of tissue-resident memory T cells (Trm)

**Fig 6:**
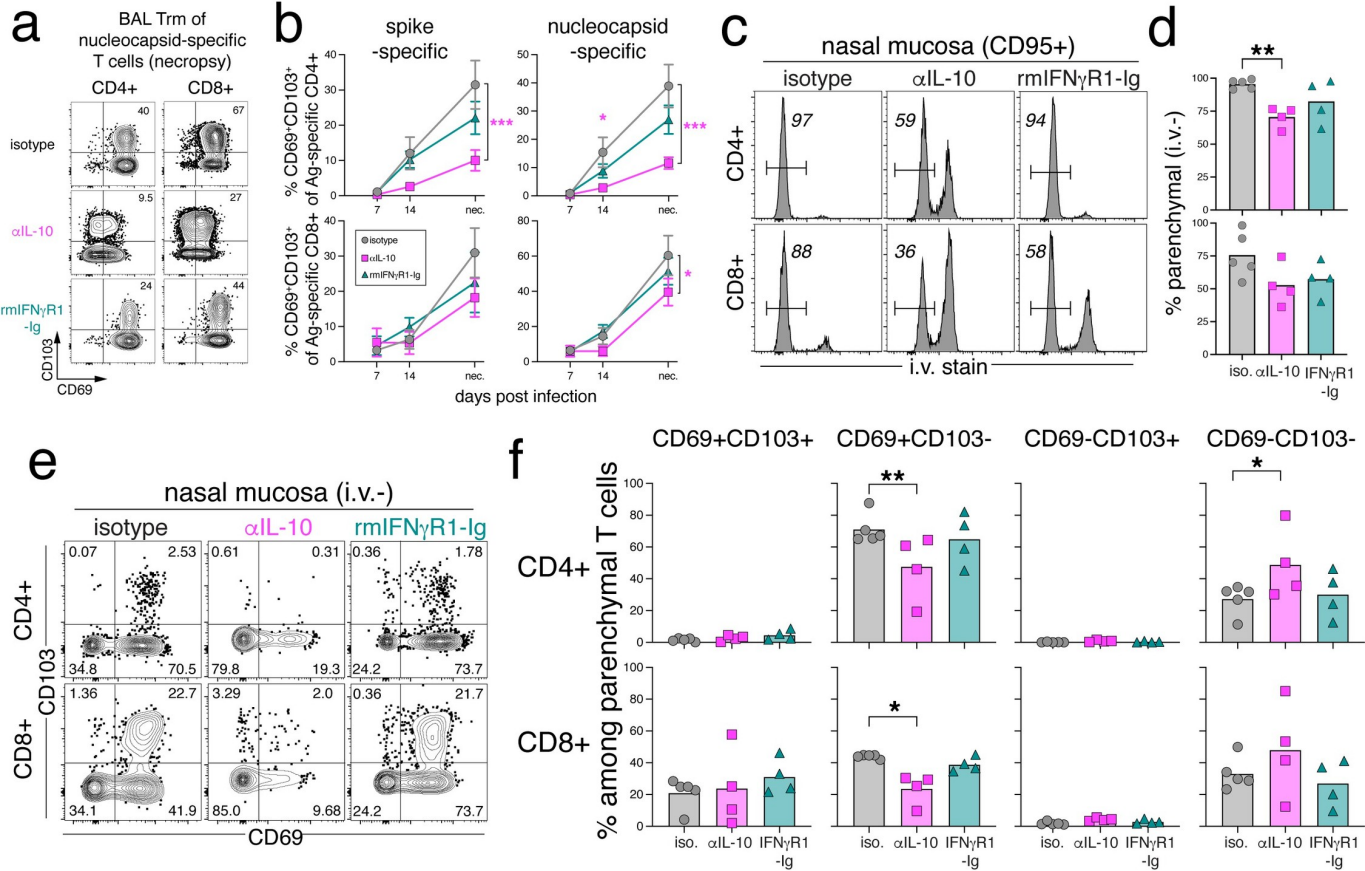
IL-10 blockade impairs the differentiation and maintenance of SARS-CoV-2-specific Trm cells in the respiratory tract. (A) Representative flow cytometry plots of CD69 and CD103 expression among nucleocapsid-specific CD4^+^ and CD8^+^ T cells at necropsy. Numbers in upper right represent the frequency of CD69^+^CD103^+^ gate. (B) Quantification of the frequency of CD69^+^CD103^+^ among spike-specific and nucleocapsid-specific CD4^+^ T cells and CD8^+^ T cells from BAL from day 7, 14, and necropsy (day 28 or 35) post-infection. Graph shows mean and SEM. (C) Representative flow cytometry plots of bulk CD4^+^95^+^ or CD8^+^95^+^ T cells with intravenous stain (i.v. stain) from the nasal mucosa of rhesus macaques infected with SARS-CoV-2 at necropsy. Numbers in plots indicate the frequency of i.v. stain negative. (D) The frequency of parenchymal (i.v.-) CD4^+^ or CD8^+^ T cells from the nasal mucosa of rhesus macaques infected with SARS-CoV-2 at necropsy. Significance calculated with 2-way ANOVA and Dunnett’s multiple comparison test. No i.v. stain data is available for animal ID: *DHNA* (αIL-10) and *DHGW* (rmIFNγR1-Ig). (E) Representative flow cytometry plots of parenchymal (i.v.-) CD4^+^ or CD8^+^ T cells expressing CD69 and CD103 from the nasal mucosa of rhesus macaques infected with SARS-CoV-2 at necropsy. Numbers in plots indicate the frequency within the quadrants. (F) Quantification of the frequency of CD69^-^CD103^-^, CD69^+^CD103^-^, CD69^-^CD103^+^, or CD69^+^CD103^+^ of parenchymal CD4^+^ or CD8^+^ T cells from the nasal mucosa of rhesus macaques infected with SARS-CoV-2 at necropsy (dpi 28 or 35). *DHNA* and *DHGW* not included, as above. Significance calculated with 2-way ANOVA and Dunnett’s multiple comparison test with isotype control.

After activation, primary effector T cells traffic to peripheral tissues and a subset of these cells differentiate into non-recirculating tissue resident memory T cells (Trm) [60]. As front-line responders, Trm are important in long-lived protection against viral and bacterial infections in the lung [61–64]. In tissues, Trm can be distinguished by the stable expression of CD69, a S1PR antagonist, and a subset of Trm also express the alpha E integrin, CD103 [65–67]. SARS-CoV-2-specific Trm have been detected in human lung autopsy specimens [68]. We examined expression of Trm markers CD69 and CD103 on S- and N-specific CD4 and CD8 T cells in the BAL after SARS-CoV-2 infection. At day 7 post-infection, when virus-specific T cells are first detected, 40–50% of SARS-CoV-2-specific CD4 and CD8 T cells in the BAL express CD69, and <10% of CD8 T cells co-expressed CD69 and CD103 (S8 Fig). At necropsy, most of the SARS-CoV-2-specific T cells in the BAL expressed CD69 and 30–60% co-expressed CD103 (Fig 6A and 6B). N-specific T cells had higher frequencies of Trm markers compared to S-specific T cells, and N-specific CD8 T cells had the highest frequency of CD69 and CD103 co-expression overall, ~60% of total, suggesting that nucleocapsid-specific CD8 T cells are highly tissue-resident (Figs 6A, 6B and S8). IL-10 blockade resulted in a delayed upregulation of Trm markers by SARS-CoV-2-specific CD4 and CD8 T cells (Figs 6A, 6B and S8). There was a 3-fold defect in the frequency of CD69/CD103 double-positive cells among S- and N-specific CD4 T cells in the BAL at 4–5 weeks post-infection in the anti-IL-10 treatment group relative to controls (Fig 6B). We also observed a decrease in CD69/CD103 double-positive Trm among N-specific CD8 T cells after IL-10 blockade, but this difference was less pronounced than was observed for CD4 T cells (Fig 6B). There were no significant differences in SARS-CoV-2-specific CD4 and CD8 Trm frequency with IFNγ blockade.

To better distinguish between tissue parenchymal and intravascular cells, intravenous antibody labelling was performed prior to necropsy of the infected macaques. In lung tissue, SARS-CoV-2-specific CD4 and CD8 T cells were preferentially localized in the parenchyma (i.v.^-^), as compared to bulk T cells, which were primarily in the lung vasculature (i.v.^+^) (S8 Fig). Of the T cells in the lung parenchyma, the majority were Trm and expressed CD69, +/- CD103 (S8C Fig). SARS-CoV-2-specific CD4 and CD8 T cells in the lung were enriched for Trm compared to non-specific T cells. Parenchymal localization and Trm marker expression by Ag-specific T cells in the lung did not differ with cytokine blockade, despite the decrease in Ag-specific Trm in the BAL with IL-10 blockade. Collectively, these data suggest that IL-10 primarily effects the expansion of SARS-CoV-2-specific CD4 and CD8 T cells in the airways and has less of an impact in the lung parenchyma.

Although SARS-CoV-2-specific T cells were not detected in the nasal mucosa of rhesus macaques, the majority of CD4 and CD8 T cells isolated from the nasal tissue were from the tissue parenchyma (i.v.^-^) (Fig 6C and 6D). In isotype control animals, the majority of the CD4 and CD8 T cells in the parenchyma of the nasal mucosa also expressed Trm markers, CD69^+^CD103^+/-^ (Fig 6E and 6F). After IL-10 blockade, there was a significant decrease in the frequency of parenchymal (i.v.^-^) CD4 T cells in the nasal mucosa. There was also significant loss of CD69 expression within the parenchymal CD4 and CD8 T cell subset in the nasal mucosa with anti-IL-10 treatment, and a subsequent enrichment for CD69-CD103- (Figs 6F and S8). Thus, IL-10 has a critical role in the *de novo* differentiation of SARS-CoV-2-specific CD4 and CD8 Trm in the airways and in maintaining Trm populations in the nasal mucosa.

## Discussion

Previous work has identified both protective and host detrimental roles for IFNγ in SARS-CoV-2. Several clinical studies have shown IFNγ levels are correlated with COVID-19 disease severity [8–18]. However, a case series by van Laarhoven and colleagues showed that for severely immunocompromised patients that were critically ill with COVID-19 administration of IFNγ was therapeutically beneficial and reduced viral loads [23]. Moreover, two recent murine model studies by Hilligan et al. and Lee et al. have shown that non-specific protection against SARS-CoV-2 induced by mycobacterial infection is mediated by IFNγ [24,25]. Here, we found that IFNγ blockade decreased the number, size, and duration of ^18^FDG avid lung lesions during SARS-CoV-2 infection in rhesus macaques. Transcriptional profiling of BAL cells revealed that IFNγ blockade also prolonged signatures of viral sensing and innate inflammation in the airway immune cells. Yet, IFNγ blockade did not result in evidence of increased viral replication in the upper or lower respiratory tract. These data suggest that while IFNγ may promote the development of pulmonary lesions during SARS-CoV-2, it could also be important in the resolution of transcriptional responses to infection. Collectively, our data along with previous studies show that the role for IFNγ in regulating protective inflammation and immunopathogenesis during SARS-CoV-2 infection is highly context dependent.

In contrast, IL-10 limited local lung inflammation induced by SARS-CoV-2 infection. IL-10 blockade increased the number, size, and overall metabolic activity of PET-avid lung lesions, as well as increased serum markers of inflammation. However, the increased inflammation in the lungs of animals receiving IL-10 blockade did not result in detectable changes in viral replication. Moreover, our transcriptional analysis detected relatively few changes in the response of BAL cells during the early phase of infection after IL-10 blockade. Instead, much of the impact of IL-10 blockade was on the development of the subsequent virus-specific T cell response. We found that IL-10 inhibits the magnitude of virus-specific CD4 and CD8 T cell clonal expansion in the circulation, lower airways, and to a lesser extent in pulmonary lymph nodes. Our data also revealed a significant role for IL-10 in promoting tissue residency of CD4 and CD8 T cells at mucosal surfaces. We found that IL-10 blockade decreased the rate at which SARS-CoV-2-specific effector CD4 and CD8 T cells differentiate into Trm within the lower airways. We also found that Trm in the nasal mucosa were decreased ~1 month after administration of anti-IL-10 blocking antibodies, indicating that IL-10 may also be important in the maintenance or retention of Trm in the nasal mucosa. While both CD4 and CD8 Trm seemed to rely on IL-10 for their formation or maintenance, CD4 Trm were the most significantly impacted by IL-10 blockade. Overall, these data strongly support the hypothesis that IL-10 plays a major role in promoting and/or maintaining the Trm phenotype of T cells at mucosal surfaces. Consistent with these results, previous studies have shown that IL-10 stimulates TGFβ production by monocytes, which is known to promote a Trm phenotype in T cells [65,69,70]. Coronavirus-specific T cells can also produce IL-10, and T cells themselves could be another source of IL-10 that regulates Trm differentiation in this model [28,29,71]. Future studies are needed to identify the predominant cellular sources of IL-10 and the downstream pathways that promote and maintain Trm cells after SARS-CoV-2 infection.

Despite the robust virus-specific T cell response in the lower airways, we failed to detect spike-specific CD4 or CD8 T cell responses in the nasal mucosa of rhesus macaques. This is consistent with our previous findings that spike- and nucleocapsid-specific T cells were not detected in the nasal mucosa on day 10 post-infection [2]. Previous reports have identified SARS-CoV-2-specific T cells in human nasal samples, and here we show that SARS-CoV-2-specific T cells were detected in about half of the human nasal sinus resection samples tested. Furthermore, SARS-CoV-2-specific Trm were detected in the nasal mucosa of infected mice, and Mtb-specific T cells were identified in the nasal mucosa of *M*. *tuberculosis*-infected macaques. It is not clear why primary SARS-CoV-2 infection fails to induce T cell responses in the nasal mucosa in rhesus macaques. Previous studies of human nasal swabs indicated that only previously mRNA vaccinated individuals with breakthrough SARS-CoV-2 infection generated SARS-CoV-2-specific immunity in the nasal mucosa [57]. Thus, a systemic prime and mucosal pull approach may be the key to generating robust immunity in the nasal mucosa [72,73]. Future experiments are needed to directly address this in macaques.

This study was not designed to directly test therapeutic approaches for treating severe COVID-19, as macaques do not develop signs of severe SARS-CoV-2 infection. NHP models that better recapitulate severe COVID-19 are needed to directly test the therapeutic potential of IFNγ blockade during SARS-CoV-2 infection. Our data also raise the possibility that providing exogenous IL-10 at the time of mucosal vaccination could promote the formation of tissue-resident memory T cells. These conclusions made herein are specific to the setting of well-controlled SARS-CoV-2 infection and mild disease. The role of IL-10 and IFNγ might differ during uncontrolled viral replication or severe COVID-19. Nevertheless, these results provide insight into the regulation of pulmonary inflammation and T cell immunity during effective immune responses to SARS-CoV-2 infection.

## Materials and methods

### Ethics statement

The Institutional Biosafety Committee approved all work with SARS-CoV-2 in the BSL-3 facility and approved all inactivation methods. All animal experiments were performed under animal study protocol LPD-25E (rhesus macaque) and LPD-24E (mouse) at the National Institutes of Health and approved by Institutional Animal Care and Use Committee (IACUC).

### Study design

The study was designed to assess the importance of the pro-inflammatory cytokine, IFNγ, and the anti-inflammatory cytokine, IL-10, early during SARS-CoV-2 infection. We set out to measure changes in lung inflammation, viral replication, and cellular immune responses against SARS-CoV-2 infection after cytokine blockade. The study had a predetermined end point of day 28–35 post-infection. The number of animals included in the study was based on previous studies assessing SARS-CoV-2 infection in non-human primates, as well as practical limitations.

### SARS-CoV-2 infection in rhesus macaques

Fifteen, male rhesus macaques, aged 2.5 to 5 years and weighing 3.5–5 kg, were divided into 3 treatment groups, isotype control, anti-IL-10, and rmIFNγR1-Ig. The animals were infected/treated in 5 waves of 3 animals per wave. Each wave consisted of one animal per treatment group. Animals were infected with 2x10^6^ TCID_50_ total of SARS-CoV-2/USA-WA-1: 1x10^6^ TCID_50_ in 3mL intratracheally, and 5x10^5^ TCID_50_ in 0.5mL intranasally in each nostril. For all procedures, animals were anesthetized with ketamine and dexmedetomidine. During anesthesia, animals were weighed and monitored for heart rate, respiratory rate, body temperature, and oxygen saturation. Glycopyrrolate and atipamezole were given for recovery from anesthesia. For data plotted longitudinally, any pre-infection timepoints were represented as day 0.

### SARS-CoV-2 infection in mice

Twelve-week-old female C57BL/6 mice on 45.1 congenic background from Taconic Biosciences were anesthetized with isoflurane and infected intranasally with 6x10^4^ TCID_50_ of SARS-CoV-2 Beta variant (B.1.351) in 30uL of sterile saline. In total, 10 mice were infected in two rounds of five mice each.

### Mycobacterium tuberculosis infection in rhesus macaques

Three male rhesus macaques aged ~3 years were infected with 30 to 50 CFU of H37Rv strain of *Mycobacterium tuberculosis* (Mtb) diluted in 2mL of sterile saline. Animals were anesthetized with ketamine and dexmedetomidine and Mtb was bronchoscopically instilled in the right lower lobe. Animals were humanely euthanized at a predetermined endpoint of 13–14 weeks after infection.

### Antibody treatment for SARS-CoV-2 infected rhesus macaques

Rhesus macaques were treated with anti-IL-10 antibody [IL-10R1LALA], rhesus interferon gamma receptor-1-Ig Fusion Protein [IFNGR-Ig], or isotype control antibody rhesus IgG1 (anti-DSP) [DSPR1], i.v. at 10mg/kg at two timepoints, one day prior to infection and 3 days after infection. All NHP antibodies used in the treatment regimen were engineered and produced by the NIH Nonhuman Primate Reagent Resource. Anti-IL-10 [IL-10R1LALA] is a rhesus recombinant antibody, with the LALA effector-silenced mutation on IgG1-kappa (NIH Nonhuman Primate Reagent Resource Cat#PR-1517, RRID AB_2716328). Rhesus interferon gamma receptor-1-Ig Fusion Protein [IFNGR-Ig], exists as a dimer and is a fusion between rhesus IFNGR1 extracellular domain and CH2-CH3 of rhesus IgG1 with a GGGS linker between IFNGR1 and Fc (NIH Nonhuman Primate Reagent Resource Cat#PR-0021, RRID AB_2895625). Control antibody rhesus IgG1 (anti-DSP) [DSPR1] is an anti-desipramine monoclonal antibody recombinant with rhesus IgG1 (NIH Nonhuman Primate Reagent Resource, Cat#PR-1117 RRID AB_2716330).

### Quantification of in vitro IL-10 and IFNγ signaling with reporter cell lines

HEK-Blue IL-10 (InvivoGen Cat#hkb-il10) and IFNγ (InvivoGen Cat#hkb-ifng) reporter cells lines were maintained in selection media per manufacturer instructions and then plated in test media (DMEM + 10% FBS, 1% Pen-Strep) at 50,000 cells per well in 180uL in a flat bottom 96-well plate. 200pg of IL-10 in 10uL was added to each well of HEK-Blue IL-10 cells. Increasing 5-fold dilutions of anti-IL-10 (2.5pg-5mg) in 10uL were added to the respective IL-10 containing wells. 40pg of IFNγ in 10uL was added to each well of HEK-Blue IFNγ cells. Increasing 2.5-fold dilutions of rmIFNγR1-Ig (2.5pg-5mg) in 10uL were added to the respective IFNγ containing wells. Control wells were plated without blockade reagents or with cytokines. Plates were incubated for 24 hours at 37°C in 5% CO_2_. The following day, QUANTI-Blue solution for detection was prepared per manufacturer’s instructions. 180uL of the QUANTI-Blue solution was added to a 96-well flat bottom plate. 20uL of the supernatant from the overnight cell culture was added to the QUANTI-Blue solution. The mixture was incubated at 37°C in 5% CO_2_ for 2 hours. The amount of cytokine signaling was determined with a color change reaction and quantified with a spectrophotometer at 650nm.

### Blood and BAL collection

Blood and BAL were collected at baseline (5–19 days prior to infection) and on days 3, 7, 14 post-infection, and at necropsy (day 28 or 35 post-infection). Blood was collected in EDTA tubes and centrifuged at 1,300g for 10 minutes at 22°C to isolate and remove plasma. The remaining blood was diluted 1:1 with 1x PBS and added to Leucosep tubes (Greiner Bio-One, Cat#227290) containing 15mL of 90% Ficoll-Paque density gradient (Cytiva Cat#17144002). Tubes were centrifuged at 1,000g for 10 minutes at 22°C. The upper layer was removed and diluted to 50mL with PBS +1% FBS, and then centrifuged at 1,600rpm for 5 minutes at 4°C. The cells were resuspended at 2x10^7^ cell/mL in X-VIVO 15 media + 10% FBS for staining or *ex vivo* stimulation. BAL was collected by instillation of 50mL of pharmaceutical-grade PBS, 10mLs at a time. BAL was then filtered through a 100um filter and centrifuged at 1,600 rpm for 5 minutes at 4°C. The cell pellet was resuspended at 2x10^7^ cell/mL in X-VIVO 15 media + 10% FBS for staining or *ex vivo* stimulation or cryopreserved in 10% DMSO + 90% FBS. Complete blood counts (CBC) were measured on an ProCyte DX (IDEXX Laboratories Inc, Westbrook, Maine) using manufacturer instruction and reagents.

### Clinical measurements

The procedures for clinical observations and measures were described previously [2]. Blood chemistries were measured at baseline and necropsy. C-Reactive Protein (CRP) was measured at baseline, 2, 3, 7, 10 days post-infection, and at necropsy and analyzed in compliance with manufactory instructions for the QuickRead go instrument with software version of 6.3.6 (AIDIAN Oy, Espoo, Finland) and QuickRead go CRP kit. The system has a range of 5–150 mg/L and values below limit of detection were plotted at 5mg/L. Fibrinogen levels were measured at baseline, 2, 3, 7, 10, 23 days post-infection and necropsy using a Satellite analyzer, kits, and control sets (Diagnostica Stago Inc, Parsippany, NJ).

### ^18^FDG-PET/CT acquisition and data analysis

Macaques were imaged using an LFER 150 PET/CT scanner (Mediso Inc, Budapest, Hungary) at baseline (6 to 20 days prior to infection), 2, 6, 10 and 22–24 days post-infection using ^18^FDG (0.5 mCi/Kg) and images of the chest were processed as previously described [2]. In each chest scan, regions of interest with abnormal density (HU > -500) and/or metabolic activity (FDG uptake > 1.5 SUV) were identified as volumes of interest (VOI) or lesions in the day 2 and/or day 6 scan. For each animal, the largest VOI across time points was transferred to the aligned PET/CT images at other time points to assess the change in lesion volume or FDG uptake, as previously described. Lesions that appeared to be continuous in the PET/CT but resulted from inflammation in more than one lung lobe (e.g., the right cranial lobe and the right middle lobe) were handled separately at necropsy and were separated in the scan using the branching of the bronchial tree as a guide. This was necessary in 2 instances, once each in DHBA and DHMC.

In this study, two additional regions were imaged: the upper abdomen including the spleen and transverse colon and the head and neck. Fused PET/CT images of the head and neck were used to estimate ^18^FDG uptake in tonsils (pharyngeal and palatine) and nasal turbinates. Similarly, to regions in the lungs, VOI were drawn on top of the fused PET/CT image for each time point of the study. In this case, only ^18^FDG uptake values were recorded. A single value was recorded for pharyngeal and palatine tonsils by creating a mask that included all of them in one VOI per time point. Both the maxilloturbinates (MT), ethmoturbinates (ET) were included in a single VOI mask per time point. ^18^FDG uptake was also recorded from a region of about 1 mL of the Trapezius muscle to normalize SUV values of tonsils and turbinates across imaging sessions. Fused PET/CT images of the lower thorax and upper abdomen were used to estimate ^18^FDG uptake in the spleen. A single VOI was created on the baseline PET/CT of the subject. Each scan thereafter was then aligned to match that of the baseline PET/CT. The baseline VOI was then copied onto each timepoint to match the volume of the original VOI of the baseline PET/CT. ^18^FDG uptake was recorded from each timepoint within the area of the spleen. ^18^FDG uptake was also recorded for muscle by the same technique as described previously [74]. The abdominal scan was not available for all subjects, so only those animals where the standard-sized VOI could be mapped on all time points are presented in Fig 1.

### Plasma cytokine analysis

Plasma cytokine levels were measured with the Luminex NHP XL Cytokine Premixed Kit (R&D Systems, Cat#FCSTM21). Samples were acquired with a MAGPIX with xPONENT software (Luminex Corporation). The background levels were determined by taking the lowest values in the standard curve for each cytokine, and this value was subtracted from each reported cytokine concentration. Values less than zero were reported as zero.

### Necropsy and intravenous stain for SARS-CoV-2 infected rhesus macaques

To assess the parenchyma localization of T cells within the highly vascularized tissues we utilized the method of intravenous antibody (i.v.) staining prior to euthanasia [75–77]. Under anesthesia but prior to euthanasia, 10mL of blood was drawn as a negative control for i.v. staining. After blood draw, 30ug/kg of anti-CD45-biotin (clone: MB4-6D6, Miltenyi) was infused for 10 minutes prior to humane euthanasia. During incubation time, BAL fluid and 60mL of blood was collected. After euthanasia, the lungs and attached airways, nasal turbinates, salivary gland, tonsil, spleen, and lymph nodes (axillary and pulmonary) were prosected. Regions of the lung that were normal or previously had abnormal HU density or FDG uptake by PET/CT analysis were individually excised from the lung, as previously described [2]. Pulmonary lymph nodes were isolated separately into two categories: those with unchanged ^18^FDG uptake or had low avidity versus those with previous ^18^FDG uptake above SUV 2.5 by PET/CT analysis were isolated separately. Tissues and lymph nodes were then divided for RNA isolation, fixation and histology, and single cell preparations for flow cytometry. No i.v. stain data is available for animal ID: *DHNA* (αIL-10) and *DHGW* (rmIFNγR1-Ig). Mtb-infected macaques were humanely euthanized as above but did not receive i.v. stain prior to necropsy.

### Necropsy and intravenous stain for SARS-CoV-2 infected mice

Prior to euthanasia mice were injected with 2ug i.v. stain anti-mouse CD45 (clone: 30-F11) BB515 (BD Cat#564590) in 300uL sterile saline. Antibody was left to circulate 3 minutes prior to euthanasia. Lungs and nasal mucosa, including nasal turbinates, were dissected after euthanasia.

### Tissue digestion for rhesus macaque tissues

All tissues were processed into single cell suspensions for *ex vivo* peptide stimulation and flow cytometry, as previously described [2]. Briefly, tissues were homogenized in gentleMACS C tubes (Miltenyi Cat#130096334). The tonsil, nasal mucosa, and lung were further digested in RPMI + 50U/mL DNase I + 1mg/mL hyaluronidase + 1mg/mL collagenase D (Roche) on a shaker at 220rpm for 45 minutes at 37°C. The digestion reaction was stopped with equal parts PBS + 20% FBS. After homogenization and/or digestion, the salivary gland, tonsil, nasal mucosa, and lung single cell suspensions were filtered through a 100um filter and then centrifuged at 1,600rpm for 5 minutes at 22°C. Cell pellets were resuspended in 40% Percoll (Sigma Cat# P1644) gradient and centrifuged at 2,000rpm for 20 minutes at 22°C. The spleen and lung were further cleared of red blood cells by resuspending cell pellets in 2mL of ACK Lysing Buffer (Quality Biologicals Cat#118-156-101) for 2 minutes at room temperature, then stopping the reaction with 10-20mL of PBS + 1% FBS. Cells were resuspended at 2x10^7^ cell/mL in RPMI media + 10% FBS for ex vivo peptide restimulation assays and flow cytometry analyses.

### Tissue digestion for mouse tissues

Dissected lungs and nasal mucosa, including nasal turbinates, were placed in 5mL of digest media (RPMI + 50U/mL DNase I + 1mg/mL hyaluronidase + 1mg/mL collagenase D (Roche)) and homogenized in gentleMACS C tubes (Miltenyi Cat#130096334) and then incubated on a shaker at 220rpm for 30 minutes at 37°C. After digestion cells were filtered through a 100um filter and the reaction was stopped with PBS+ 20% FBS. The single cell suspension was then spun at 1,600rpm for 5 minutes. The cell pelleted was resuspended in 5mL of 37% Percoll (Sigma Cat# P1644) gradient and centrifuged at 2,000rpm for 20 minutes at 22°C without brake. The cell pellet was resuspended in 2mL of ACK Lysing Buffer (Quality Biologicals Cat#118-156-101) for 2 minutes at room temperature, then stopping the reaction with 10mL of PBS + 1%FBS. Cells were resuspended at 2x10^7^ cell/mL in X-VIVO 15 media + 10% FBS for flow cytometry analyses.

### Tissue digestion for human nasal tissue

Discarded surgical material from individuals undergoing nasal debridement for allergic and obstructive complications unrelated to SARS-CoV-2 were collected from September 2022 to September 2023. Samples were removed of all identifying patient information prior to receipt by the research lab members and were not identifiable to patients, surgeons, or study authors. Only de-identified aggregate data generated by the research lab were shared with the co-authors and surgical team. Accordingly, this study was deemed exempted from IRB review. Samples were obtained fresh, transported in PBS, and isolated for single cells the same day. The material ranged in size from 2-10mm across. The tissue was placed in 5mL of digest media (RPMI + 50U/mL DNase I + 1mg/mL hyaluronidase + 1mg/mL collagenase D (Roche)) and homogenized in gentleMACS C tubes (Miltenyi Cat#130096334) and then incubated on a shaker at 220rpm for 30 minutes at 37°C. After digestion cells were filtered through a 100um filter and the reaction was stopped with PBS+ 20% FBS. The single cell suspension was then spun at 1,600rpm for 5 minutes. The cell pelleted was resuspended in 5mL of 37% Percoll (Sigma Cat# P1644) gradient and centrifuged at 2,000rpm for 20 minutes at 22°C without brake. Cells were resuspended at 2x10^7^ cell/mL in X-VIVO 15 media + 10% FBS for ex vivo peptide restimulation assays and flow cytometry analyses. A total of n = 14 viable samples were obtained and assessed for SARS-CoV-2 specific responses and of these, 5 were also included in the CMV/EBV specific assessments.

### Peptide stimulation assay

Single cell suspensions were plated at 2x10^6^ cells per well in round bottom 96 well plates in 200uL of X-VIVO 15 media, supplemented with 10% FBS, Brefeldin at 1000x (Invitrogen Cat#00-4506-51), Monensin at 1000x (Invitrogen Cat#00-4505-51), and with or without peptide pools at 1ug/mL. Plates were incubated at 37°C + 5% CO_2_ for 6 hours. The spike peptide pool consisted of Peptivator SARS-CoV-2 Prot_S1 (Miltenyi Cat#130-127-048) and Peptivator SARS-CoV-2 Prot_S (Miltenyi Cat#130-127-953). The nucleocapsid peptide pool consisted of Peptivator SARS-CoV-2 Prot_N (Miltenyi Cat# 130-126-699). The membrane peptide pool consisted of Peptivator SARS-CoV-2 Prot_M (Miltenyi Cat# 130-126-703). CD4 megapool consisted of CD4_S_MP and CD4_R_MP, and CD8 megapool consisted of CD8_MP_A and CD8_MP_B, as described [55]. Human nasal samples were additionally stimulated with the Peptivator SARS-CoV-2 Prot_S Complete (Miltenyi Cat#130-127-953), Peptivator SARS-CoV-2 Prot_S B.1.617.2 (Miltenyi Cat#130-128-763), CMV megapool, and EBV megapool [59]. For rhesus macaque T cell panels, CD107a (H4A3) Alexa Fluor 647 (Biolegend Cat#328612) at 1:50, CD107b (H4B4) Alexa Fluor 647 (Biolegend Cat#354312) at 1:50 was added to the stimulation cocktails. For the human T cell panels, CD107a (H4A3) BV711 (Biolegend Cat#328640) at 1:50 was added to the stimulation cocktails. The frequency of Ag-specific cells was calculated based on the frequency of IFNγ and/or TNF+ cytokine producing CD4 or CD8 T cells in the stimulated wells minus the frequency in the unstimulated wells, as previously described [2]. The Mtb300 peptide pool consisted of MHC-I and MHC-II peptides from Mtb at 1 and 2ug/mL respectively. The frequency Mtb-specific cells was calculated based on the frequency of IFNγ+/TNF+ cytokine producing CD4 or CD8 T cells. Significance was calculated based on the difference between matched stimulated and unstimulated samples.

### Flow cytometry and antibody staining

Cells for B cell staining panels were resuspended in 50uL Human Fc-Block (BD Cat#564220) diluted to 1:500 in PBS + 1%FBS and incubated for 30 minutes at 4°C prior to washing and surface staining. Cells for MAIT cell staining panels were resuspended in 40uL RMPI + Dasatinib at 1000x, Brefeldin at 1000x, and Monsensin at 1000x, and incubated for 10 minutes at 37°C, then 10uL of rhesus macaque MR1 tetramer APC (NIH tetramer core facility) was added and incubated for an additional hour at 37°C. For surface staining, cells were spun down and resuspended in 50uL of antibody cocktail diluted in PBS +1%FBS +10x Brilliant Violet Staining Buffer Plus (BD Cat#566385), and incubated at 20 minutes at 4°C. For mouse spike-tetramer staining, H-2K(b) SARS-CoV-2 S_539-546_ (VNFNFNGL) tetramer in PE (NIH tetramer core facility) was added at 1:100 in the surface staining cocktail and incubated for 30 minutes at 4°C.

After surface staining, cells were washed 3 times in PBS + 1% FBS, and resuspended in 100uL of eBioscience Intracellular Fixation & Permeabilization Buffer (Thermo Cat# 88-8824-00) and incubated for 16 hours at 4°C. After fixation, cells were centrifuged at 2,200rpm for 5 minutes at 4°C without brake and washed once with eBioscience Permeabilization Buffer. Cells were resuspended in 50uL intracellular stains diluted in eBioscience Permeabilization Buffer +10x Brilliant Violet Staining Buffer Plus, and stained for 30 minutes at 4°C. After staining cells were washed with eBioscience Permeabilization Buffer 2x and resuspended in PBS + 1% FBS + 0.05% Sodium Azide for analysis on the BD Symphony or the Cytek Aurora platforms.

Non-human primate T cell panel included the following surface-stained antibodies: CD3 (SP34-2) BUV805 (BD Cat#568354), CD4 (SK3) BUV496 (BD Cat#612936), CD8a (RPA-T8) BV510 (Biolegend Cat#301048), CD28 (CD28.2) PE/Dazzle 594 (Biolegend Cat#302942), CD95 (DX2) BUV737 (BD Cat#612790), CD69 (FN50) FITC (Biolegend Cat#310904), CD103 (B-Ly7) PE (Invitrogen Cat#12-1038-42), and Fixable Viability Dye eFluor780 (eBioscience Cat#65-0865-14); and the following intracellular-stained antibodies: IFNγ (4S.B3) BV711 (Biolegend Cat#502540), TNF (Mab11) BUV395 (BD Cat#563996), and Ki67 (B56) PE-Cy7 (BD Cat#561283). At Necropsy, CD69 was stained in BV785 (Biolegend Cat#310932) and Streptavidin FITC (Biolegend Cat#405202), which was used to detect the i.v. stain.

Non-human primate B cell panel included the following surface-stained antibodies: CD20 (2H7) BV785 (Biolegend Cat#302356), HLA-DR (L243) BV605 (Biolegend Cat#307640), CD95 (DX2) BV711 (BD Cat#563132), IgD (Polyclonal) FITC (Southern Biotech Cat#2030–02), IgG (G18-145) APC (BD Cat#550931), and Fixable Viability Dye eFluor780 (eBioscience Cat#65-0865-14); and the following intracellular antibodies: BCL-6 (K112-91) BV421 (BD Cat#563363), and Ki67 (B56) PE-Cy7 (BD Cat#561283). At necropsy, Streptavidin BUV805 was used to detect the i.v. stain.

Non-human primate donor-unrestricted T cell panel included the following surface-stained antibodies: CD3 (SP34-2) BUV805 (BD Cat#568354), CD4 (SK3) BUV737 (BD Cat#612748), CD8a (RPA-T8) BV510 (Biolegend Cat#301048), CD8b (SIDI8BEE) PE (Invitrogen Cat#12-5273-42, NKG2A (Z199) PE-Cy7 (Beckman_Coulter Cat#B10246), TCR γ/δ (B1) PE/Dazzle 594 (Biolegend Cat#331226), CD56 (NCAM16.2) BUV496 (BD Cat#750479), CD16 (3G8)_BV650 (BD Cat#563692), TCR Vα7.2 (3C10) BV711 (Biolegend Cat#351732), and Fixable Viability Dye eFluor780 (eBioscience Cat#65-0865-14); and the following intracellular antibodies: Granzyme B (GB11) FITC (Biolegend Cat#515403), Ki67 (B56) BV451 (BD Cat#562899). At necropsy, Streptavidin BUV395 (BD Cat#564176) was used to detect the i.v. stain prior to tetramer staining.

Mouse T cell panel included the following surface-stained antibodies: CD4 (GK1.5) BUV395 (BD Cat#565974), CD8a (53–6.7) BUV805 (BD Cat#564920), CD44 (IM7) BV785 (Biolegend Cat#103059), KLRG1 (2F1/KLRG1) BV510 (Biolegend Cat#138421), CD69 (H1.2F3) BUV737 (BD Cat#612793), CD103 (2E7) BV421 (Biolegend Cat#121422), and Fixable Viability Dye eFluor780 (eBioscience Cat#65-0865-14); and the following intracellular antibody: Foxp3 (MF-14) Alexa Fluor 700 (Biolegend Cat#126422)

For the human T cell panel, cells were incubated with LIVE/DEAD Fixable Near-IR (Invitrogen Cat#L34976) diluted 1:1000 in PBS for 15 minutes prior to surface staining. Human T cell surface stain included the following antibodies: CD3 (SP34-2) BUV805 (BD Cat#568354), CD8a (RPA-T8) BUV563 (BD Cat#612914), CD4 (OKT4) BB700 (BD Cat#566829), CD28 (CD28.2) BV480 (BD Cat#566110), and CD95 (DX2) APC/Fire 810 (Biolegend Cat#305663). Human intracellular antibodies included the following: TNF (Mab11) BUV395 (BD Cat#563996), IFNg (B27) FITC (Biolegend Cat#506504), Foxp3 (206D) PE/Dazzle 594 (Biolegend Cat#320126).

### Viral RNA quantification

Nasal and throat swabs were collected at the indicated timepoints (Fig 1). Swabs were placed in 1mL of Viral Transport Media (1x HBSS, 2% FBS, 100ug/mL Gentamicin, and 0.5ug/mL amphotericin B) and processed as previously described [2]. Liquid samples, such as swab media, BAL fluid, or plasma, were processed for RNA using using QIAmp Viral RNA mini kit (Qiagen Cat# 52906) and were eluted in 50uL RNase Free water. At necropsy, whole tissues were placed in 1mL RNAlater media (Sigma Cat# R0901) and stored at 4°C overnight and then stored at -80°C until RNA processing. Tissues were thawed and tissue pieces were transferred to clean 2mL tubes containing 600uL RLT Buffer from the RNeasy RNA Mini kit (Qiagen # 74136) and 2mm beads. Tissues were homogenized with the Precellys Tissue Homogenizer. After homogenization tissue RNA was further processed using the RNeasy Plus Mini kit and RNA eluted in 50uL RNase Free water. Eluted RNA was stored at -80°C long-term.

RT-qPCR reactions for genomic and subgenomic RNA of the N gene of SARS-CoV-2 were performed on all extracted RNA samples, as previously described [2]. The total reaction volume was 12.5uL for each sample and control, which contained 2.5uL of eluted RNA, 3.25uL Taqpath 1-step RT-qPCR Master Mix (Thermo Cat#A15299), primers at 500nM, probes at 125-200nM, and the remaining volume as RNase free water. N gene subgenomic RNA was detected using Forward Leader sequence primer (5’-CGA TCT CTT GTA GAT CTG TTC TC-3’), sgN Reverse (5′-GGT GAA CCA AGA CGC AGT AT-3’), and sgN Probe (5′-[FAM]-TAA CCA GAA TGG AGA ACG CAG TGG G-[BHQ1]-3′,) at 200nM, all custom made from Eurofins. All samples were tested for RNA integrity using the 2019-nCoV RUO Kit for RNase P, containing CDC RNAse P Forward Primer (5’-AGA TTT GGA CCT GCG AGC G-3’), CDC RNAse P Reverse Primer (5’-GAG CGG CTG TCT CCA CAA GT-3’), and CDC RNAse P Probe (5’-[FAM]-TTC TGA CCT GAA GGC TCT GCG CG-[BHQ]-1-3’). Prepared reactions were read on a QuantStudio 7 Flex Real-Time PCR System, 384-well format (Applied Biosystems Cat# 4485701). Cycling conditions: Initial: 25°C for 2 minutes, 50°C for 15 minutes, and 95°C for 2 minutes, cycling: 95°C for 3 seconds, 60°C for 30 seconds, for 40 cycles. Copies per/mL or copies/gram were calculated based on standard curves generated for each run using an RNA standard of known quantity. The limit of detection was based on the CT limit of detection from the standard curve in each run. For subgenomic RNA cutoff CT<37 was used.

### Anti-Spike/RBD antibody titers

Anti-SARS-CoV-2 IgG, IgA and IgM antibody titers were quantified with previously frozen plasma samples. Plasma IgG and IgA titers were determined in a dual IgG/IgA assay, with IgG detected by an enhanced chemiluminescent (ECL) based assay and IgA detected by a dissociation-enhanced lanthanide fluorescent immunoassay based on time-resolved fluorescence (DELFIA-TRF, Perkin-Elmer). Specifically, 96-well plates (PerkinElmer Cat#AAAND-0001) were coated overnight at 4°C with 100uL of Carbonate-Bicarbonate Buffer (Sigma Cat#SRE0034-1L) containing prefusion-stabilized spike (S-2P) at 1ug/mL or RBD at 2ug/mL, as previously described [78]. The following day, plates were washed with Wash Buffer (1x DPBS +0.1% IGEPAL CA-630 (Sigma catalog 18896-100ml)) using a plate washer and blocked overnight at 4°C with 250uL of Blocking Buffer (1x DPBS +5% dry milk). Plasma samples were thawed, heat-inactivated, and centrifuged at 2,000rpm for 5 minutes to pellet debris, and the supernatant was transferred to a new tube. Plasma was then diluted 1:100 in Dilution Buffer (1x DPBS +5% dry milk +0.2% IGEPAL CA-360). Three-fold serial dilutions were performed across columns in a 96 well plate and 100uL of diluted samples were added to the coated plates. Plates were incubated for 1 hour at room temp on a rotating shaker. After incubation, plates were washed 3 times with a plate washer. 100uL of the secondary antibody mixture consisting of Goat anti-monkey IgG(H+L)-HRP (ThermoFisher Cat# #PA1.8463) at a 1:10,000 dilution and Goat anti-monkey IgA-Biotin (Alpha Diagnostic International Cat#70049) at a 1:5,000 dilution in Dilution Buffer was added. Plates were incubated for 1 hour at room temp on a rotating shaker. After incubation, plates were washed 3 times with a plate washer, and 100uL of Streptavidin-Europium (PerkinElmer Cat#1244–360) diluted 1:2,000 in PBS +0.2% IGEPAL CA-630 was added. Plates were incubated for 1 hour at room temp on a rotating shaker. Plates were washed 3 times with plate washer, and 50uL of HRP substrate (Pierce ECL Substrate, Thermo Cat#32106) was added. After a 10 minute-incubation, HRP-mediated ECL luminescence was read using a Synergy Neo2 HTS plate reader (BioTek) to detect IgG. Then the ECL substrate was removed, and the plates were washed 3 times. After the last wash, 100uL of DELFIA Enhancement Solution (Perkin-Elmer Cat#4001–0010) was added to generate Europium-based fluorescence. After a 20 minute-incubation, time-resolved fluorescence was read using the Synergy reader to detect IgA. Plasma was analyzed in duplicate plates, and the average reading for each dilution was taken, subtracting the average from blank wells. The cutoff was set at the blank average +3 standard deviations. The titers were determined by interpolating the cutoff on sigmoid standard curve. IgM titers were measured as described for IgA, using secondary goat anti-monkey IgM-biotin (Brookwoodbiomedical Cat#1152) 1:5,000 diluted in Dilution buffer, in a stand-alone DELFIA TRF assay.

### Live virus neutralization assay

Live-virus neutralization assays were performed on Vero-E6 cells stably expressing human TMPRSS2 (Vero-E6T2), as previously described [2]. One day before the assay, cells were plated in 12-well plates (Falcon Cat#353043) at a density of 0.4 million cells per well in 2mL D10+ medium (DMEM +10% FBS, 1X Glutamax, 1X Anti-Anti (Gibco) and 250ug/mL Hygromycin B (InVivoGen)). The day of the assay, 12-well plates were washed out of selection media with D2 medium (DMEM + 2%FBS, 1X Glutamax). Frozen plasma samples from baseline, says 7, 14, and necropsy (d28-35) post-infection were thawed and inactivated at 56°C for 30 minutes. Samples were then diluted 1:5 and further 2-fold serially diluted. Dilutions were incubated with equivalent volume of 40–50 PFU of SARS-CoV-2 USA-WA1/2020 virus in D2 media for 1 hour at 37°C. After incubating, 100uL of virus/plasma mix + 300uL of D2 media was added to Vero-E6T2 cells, in duplicate. The cells were then incubated for 1 hour at 37°C +5% CO_2_, with occasional gentle agitation. After this incubation, 1.5mL of DMEM +0.6% methylcellulose per well was added. Plates were then incubated for 66–72 hours at 37°C +5% CO_2_. The media was then gently removed and 1mL of Crystal Violet +5% Ethanol and 3% formalin was added, and stained for 20 minutes at room temp. The cell layer was washed with deionized water and then air dried. Plates were scanned and counted for plaques. Percent inhibition was calculated based on plaques at each dilution compared to viral samples incubated without plasma. IC_50_ of percent inhibition was calculated based on non-linear regression.

### Transcriptional analysis of BAL cells with Nanostring

Cryopreserved BAL cells from pre-infection (d0) and days 3, 7, 14, and 28–35 post infection (necropsy) were thawed and homogenized using Qiagen RLT buffer Plus (Cat #1053393) and QIAShredder Columns (Cat # 79656). RNA was isolated using the Qiagen RNeasy Mini kit (Cat # 74134) according to manufacturer’s instructions and eluted in 25uL of RNase free water. RNA was concentrated to 25-50ng/uL using Eppendorf Vacufuge plus centrifuge concentrator. RNA quality was confirmed with Agilent TapeStation. RNA was sent to the National Cancer Institute (NCI) Center for Cancer Research Genomics Core at the Frederick National Laboratory. Digital mRNA gene expression analysis was performed with the Nanostring nCounter NHP Immunology panel V2 (Cat #115000276). Raw read counts were obtained from RCC files using the nSolver 4.0 Data Analysis software and the NS_NHP_Immunology_V2.0 rlf file references included with the nCounter NHP Immunology panel V2 kit. After quantification, one sample, DHMM day 14, was excluded due to low concentration and poor-quality RNA.

Each genes’ molecular degree of perturbation (MDP) overtime was assessed, regardless of treatment groups, using the mdp package (Lever M, Russo P, Nakaya H (2023). mdp: Molecular Degree of Perturbation calculates scores for transcriptome data samples based on their perturbation from controls. doi: 10.18129/B9.bioc.mdp, R package version 1.22.0, https://bioconductor.org/packages/mdp). Day 0 (time 0) was used as a reference to calculate the MDP in the other timepoints. The MDP scores were used to classify the genes by time points into variability classes, which were defined as 1) Low variability genes with MDP score < 2; 2) mid variability genes with MDP score > 2 and < 5; 3) high variability genes with MDP score > 5. The overall genes’ classification was defined by the *three periods’ classification mean*, after assigning values of 1, 2 and 3 for Low variability, Mid variability, and High variability, respectively. Genes with overall high variability had the three periods’ mean > = 1.9, mid variability’s mean > 1.1 and <1.9and low variability’s mean was < = 1.1. A total of 569 genes had Low variability overtime, 202 had Mid variability overtime, and 13 had High variability overtime. Mid and High variability genes were considered upregulated after infection and grouped together for further analysis. A heatmap was generated using z-scores of variable genes and 5 clusters of genes were defined by k-means clustering. Enriched gene ontology (GO) pathways were determined for each cluster using EnrichR [37]. The top 10 most important genes for each cluster have been identified by the random forest machine learning algorithm [79], after sorting by gini index, which is related to the tree’s highest homogeneity regarding the groups. Differential expression was calculated between treatment groups compared to control, using the NanoStringDiff package (DOI: 10.18129/B9.bioc.NanoStringDiff). Genes with fold2 change >1.5 and false discovery rate <0.05 were considered differentially expressed. Median normalized gene counts were used to plot expression for individual genes over time.

### Statistical analysis

Data were analyzed using a 2way ANOVA with a Sidak’s multiple comparison test, a Tukey’s multiple comparison test, or a Dunnett’s multiple comparison test, a one-way ANOVA using a Dunnett’s multiple comparison test, or a two-tailed paired t-test. Tests used are indicated in figure legends. For all statistical analysis p<0.05 for the given test is considered significant: * p<0.05, ** p<0.01, *** p<0.001, **** p<0.0001.

## Supporting information

S1 Table Gene Ontology (GO) terms associated with SARS-CoV-2 responsive gene clusters. Gene Ontology (GO) terms that were statistically significant in association with genes defined in transcriptomic analysis of SARS-CoV-2 responsive gene cluster 1–5 and used in Fig 3C. Data was calculated with EnrichR, see Materials and Methods.
(XLSX)

S1 Fig *In vitro* blockade of IL-10 and IFNγ signaling with anti-IL-10 and rmIFNγR1-Ig reagents. (A) Quantification of the relative of the amount of *in vitro* IFNγ signaling by rhesus macaque IFNγ that is inhibited by varying amounts of the rmIFNγR1-Ig reagent used in the *in vivo* studies. Dilutions of rmIFNγR1-Ig range from 1ng-0.1mg. (B) Quantification of relative of the amount of *in vitro* IL-10 signaling inhibited by varying amounts of the anti-IL-10 reagent used in the in vivo studies. Dilutions of anti-IL-10 range from 1ng-0.05mg. The amount of cytokine signaling in *A* and *B* was evaluated with InvivoGen HEK-Blue reporter cells lines and relative levels enumerated with a spectrophotometer at 650nm. (C-D) Plasma concentrations of IFNγ in *C* and IL-10 in *D* at the indicated timepoints after SARS-CoV-2 infection as measured by Luminex assay in pg per mL of plasma with background values subtracted.
(EPS)

S2 Fig ^18^FDG-PET/CT imaging of lungs of SARS-CoV-2 infected rhesus macaques. (A) 3D rendering of lung ^18^FDG-PET/CT images from baseline, day 2, 6, 10, and 21–24 post infection. Animal IDs are embedded in white. Animals are grouped by treatment. (B) Quantification of mean lung lesion density in Hounsfield Units (HU) (y-axis) and volume of individual lesions (size of dot) over time, based on VOI defined at day 2 or 6 post infection. Significance between groups at each timepoint, and between day 2 and day 6 within groups, was determined by 2-way ANOVA and Tukey’s multiple comparison test.
(TIF)

S3 Fig Expression of inflammatory genes is prolonged with IFNγ blockade. Median normalized counts of mRNA from BAL immune cells after SARS-CoV-2 infection. The genes shown are 5 of the top 10 most important genes in each gene cluster, as reported in Fig 3. The significance and differential expression for each treatment group compared to controls is reported in Fig 3.
(EPS)

S4 Fig NK cells responses to SARS-CoV-2 infection. (A) Representative flow cytometry plots of NK cell gating strategy (NKGA2+) and sub-setting (CD56 vs. CD16) in PBMCs and BAL from DHMM (isotype), DHNA (anti-IL-10), and DHGW (rmIFNγR1-Ig) at day 3 after SARS-CoV-2 infection. (B) Quantification of NK cell subset responses as a frequency of total live lymphocytes at baseline, days 3, 7, 14 post infection and necropsy (days 28–35 post-infection). NKG2A+: CD56-/CD16+ (blue), CD56+/CD16- (green), CD56+/CD16+ (orange), CD56-/CD16- (grey). Lines are mean and error bars are SEM. Significance calculated with 2-way ANOVA and Dunnett’s multiple comparison test. (C) Representative flow cytometry plots of Ki67 expression by NK cell subsets in PBMCs and BAL from DHMM, DHNA, and DHGW at day 7 post-infection. (D) Quantification of the frequency of Ki67+ of NKG2A+: CD56-/CD16+, CD56+/CD16-, CD56+/CD16+, and CD56-/CD16- subsets at baseline days 3, 7, 14, and 28/35 post-infection in PBMC (top) and BAL (bottom). Significance calculated with 2-way ANOVA and Dunnett’s multiple comparison test. (E) Representative flow cytometry plots of Granzyme B expression by NK cell subsets in PBMCs and BAL from DHMM, DHNA, and DHGW at day 7 post-infection. (F) Quantification of the frequency of Granzyme B+ of NKG2A+: CD56-/CD16+, CD56+/CD16-, CD56+/CD16+, and CD56-/CD16- subsets at baseline days 3, 7, 14, and 28/35 post-infection in PBMC (top) and BAL (bottom).
(EPS)

S5 Fig MAIT cell responses to SARS-CoV-2 infection. (A) Representative flow cytometry plots of MR-1 tetramer staining vs. Ki67 from PBMCs and BAL from DHMM at baseline days 3, 7, 14, and 28/35 post-infection. Plots gated on CD8α+/CD8β+/γδTCR-. (B) Quantification of MAIT cell frequency as percentage of CD8αβ+/γδTCR- in PBMCs and BAL. Each animal is represented as a point and the mean as a line for each treatment group. (C) Quantification of % Ki67+ of MAIT cells in PBMCs and BAL. (D) Quantification of MAIT cell frequency as percentage of CD8αβ+/γδTCR- in the spleen, axillary lymph node (axLN), normal pulmonary lymph node (norm. pulm. LN), previously hot pulmonary lymph node, tonsil, and lung at day 28 or 35 necropsy.
(EPS)

S6 Fig B cell and antibody are minimally impacted by IL-10 and IFNγ blockade. (A) Frequency of CD20+ B cells in PBMCs and BAL as a percentage of total live lymphocytes at baseline and days 3, 7, 14, and 28/35 post-infection necropsy (nec.). Lines are mean and error bars are SEM. (B) Frequency of total B cells from PBMCs, BAL, lung, tonsil, spleen, axillary lymph node (axLN), and previously hot pulmonary lymph nodes (prev. hot pulm. LN) as a percentage of total live lymphocytes at day 28/35 post-infection necropsy. (C) Representative flow cytometry plots of gating strategy for germinal center B cells from animals DHDI, DHGJ, and DHEX isolated from previously PET hot pulmonary lymph nodes. Gates in plots show frequency of germinal center B cells as a frequency of CD20+ total B cells. (D) Quantification of germinal center B cells as a frequency of total B cells from the indicated tissues as necropsy. (E) Plasma anti-spike (left graphs) and anti-RBD (right graphs) antibody titers of the indicated isotypes: IgM, IgA, and IgG, in log10 endpoint titer at baseline and days 3, 7, 14, and 28 or 35 post-infection necropsy. Each animal is represented as a point and the mean as a line for each treatment group. Significance calculated with 2-way ANOVA and Tukey’s multiple comparison test. Significance not reported for comparisons with <3 data points. (F) Live-virus plasma neutralization titer at baseline and days 3, 7, 14, and 28 or 35 post-infection necropsy. Titer calculated as a 50 percent plaque reduction neutralization titer (PRNT50) of negative control sera. Each animal is represented as a point and the mean as a line for each treatment group. (G) Correlation analysis of neutralization titer and anti-RBD IgM, IgG, or IgA titer at 28 or 35 post-infection necropsy using simple linear regression with r-squared of goodness of fit and p-value of non-zero slope reported.
(EPS)

S7 Fig Antigen-specific T cells are present in the nasal mucosa of Mtb infected rhesus macaques. (A) (Left) Representative flow cytometry plots of CD4+95+ or CD8+95+ T cells responding to Mtb300 peptide pool from the nasal mucosa of rhesus macaques infected with *Mycobacterium tuberculosis* at necropsy (14 weeks post-infection). Numbers in plots are the frequency of the quadrants. The green shading represents the frequency of IFNγ+TNF+ in the Mtb300 stimulated sample. (Right) Representative flow cytometry plots of CD69 and CD103 expression by antigen-specific (green dots) and non-specific (grey dots) CD4+CD95+ or CD8+CD95+ T cells responding to Mtb300 peptide stimulation in the nasal mucosa of rhesus macaques infected with *Mycobacterium tuberculosis*. (B) Quantification of frequency of IFNγ+TNF+ CD4+95+ or CD8+95+ T cells from Mtb300 stimulated and unstimulated samples from the nasal mucosa of rhesus macaques infected with *Mycobacterium tuberculosis*. Significance calculated with individual t-test.
(EPS)

S8 Fig Kinetics of CD69 and CD103 expression on SARS-CoV-2-specific T cells in BAL and lung. (A, C) Representative flow cytometry plots of CD69 and CD103 expression among spike-specific and nucleocapsid-specific CD4+ and CD8+ T cells from bronchoalveolar lavage (BAL) at day 7, 14, and necropsy (day 28 or 35) post-infection. Numbers in the upper right represent the frequency of CD69+CD103+. (B, D) Quantification of the frequency of CD69 or CD103 expressing subsets among spike-specific (B) and nucleocapsid-specific (D) CD4+ T cells and CD8+ T cells from BAL at day 7, 14, and necropsy (day 28 or 35) post-infection. Graph shows mean and SEM. CD69-CD103- (grey bars), CD69+CD103- (dark blue bars), CD69-CD103+ (green bars), and CD69+CD103+ (turquoise bars). (E) Representative flow cytometry plots of antigen-specific (light blue) and bulk non-specific (grey) CD4+95+ (top row) and CD8+95+ (bottom row) T cells from concatenated lung tissue samples from DHNC isotype control animal at necropsy. Antigen-specific and bulk T cell populations were subsetted for the frequency of intravenous antibody stain (i.v. stain) negative cells, and then among i.v. stain negative cells the frequency of CD69+ and CD103+. Numbers in gates are the indicated frequencies within the gates. (F) Quantification of the frequency of lung parenchymal localization determined by negative i.v. staining, among bulk and antigen-specific CD4+ and CD8+ T cells from concatenated lung samples at necropsy. Graphs are plotted as mean and individual animals as points. Significance determined by 2-way ANOVA with Dunnett’s multiple comparison test. No iv stain data is available for animal ID: *DHNA* (αIL-10) and *DHGW* (rmIFNγR1-Ig). (G) Quantification of the frequency of Trm markers: CD69-CD103- (grey bars), CD69+CD103- (dark blue bars), CD69-CD103+ (green bars), and CD69+CD103+ (turquoise bars) among parenchymal antigen-specific and bulk CD4+ and CD8+ T cells from concatenated lung samples at necropsy. Graphs represent mean and SEM. Significance determined by 2-way ANOVA with Dunnett’s multiple comparison test. Only samples with >35 events for antigen-specific cells were used for phenotypic analysis.
(EPS)
