# Supplementary figures and images for: IL-10 suppresses T cell expansion while promoting tissue-resident memory cell formation during SARS-CoV-2 infection in rhesus macaques
Source: PLoS Pathog. 2024 Jul 1;20(7):e1012339. doi: 10.1371/journal.ppat.1012339 (PMC11244803; doi:10.1371/journal.ppat.1012339)

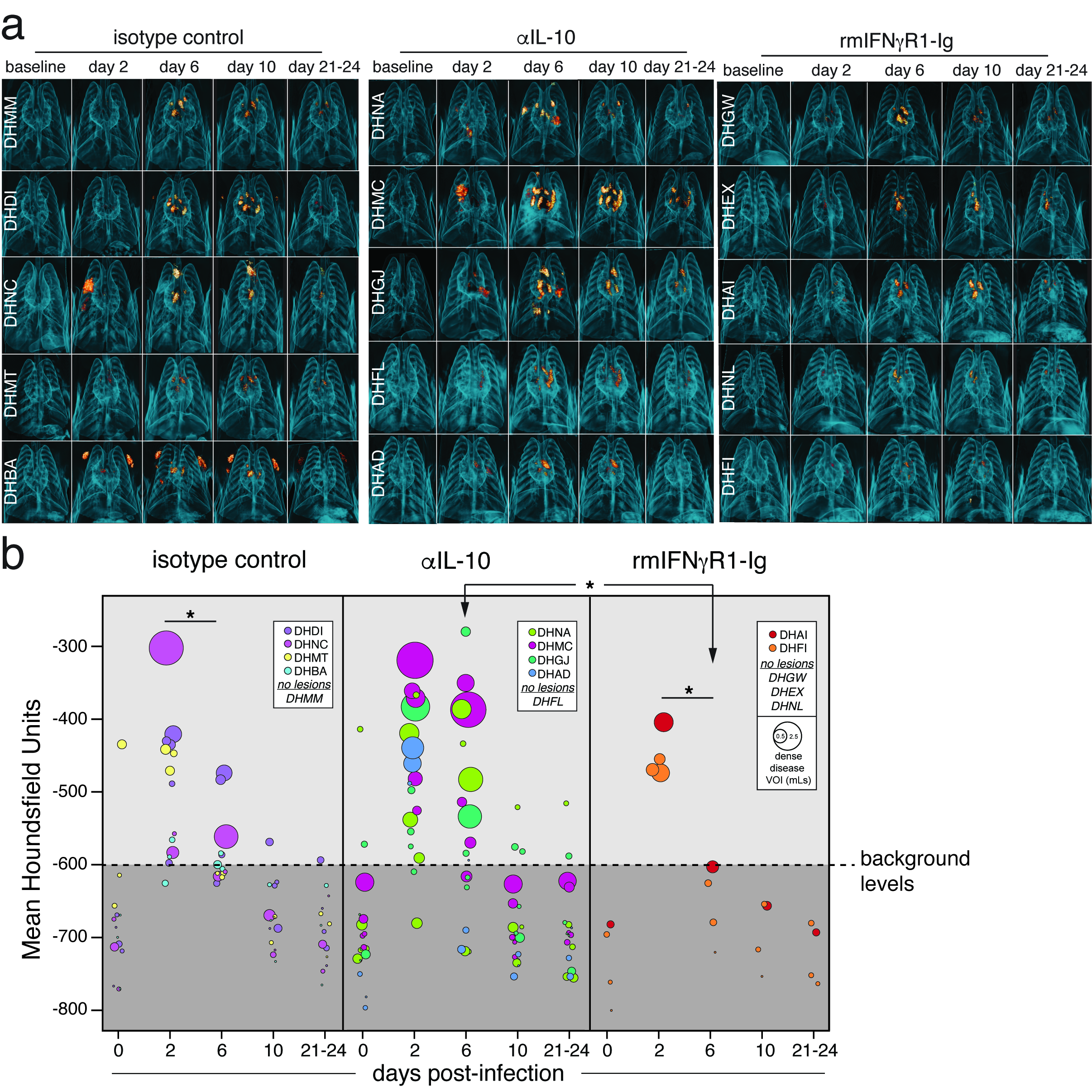

Supplement: S2 Fig — (A) 3D rendering of lung 18FDG-PET/CT images from baseline, day 2, 6, 10, and 21–24 post infection. Animal IDs are embedded in white. Animals are grouped by treatment. (B) Quantification of mean lung lesion density in Hounsfield Units (HU) (y-axis) and volume of individual lesions (size of dot) over time, based on VOI defined at day 2 or 6 post infection. Significance between groups at each timepoint, and between day 2 and day 6 within groups, was determined by 2-way ANOVA and Tukey’s multiple comparison test. (TIF) [file ppat.1012339.s003.tif]
